# AI-enabled HRM systems in business organizations: effects on workforce outcomes and the roles of trust and privacy

**DOI:** 10.3389/frai.2026.1847241

**Published:** 2026-09-10

**Authors:** Hriday Chandra Shil, S. K. Md. Anik Hassan Rabby, Md Mostafizur Rahman, Nashita Mumtahina, Md Anamul Islam, Faria Zafreen, Asraful Islam, Md Al Fassi

**Affiliations:** 1 Department of Management Studies, Comilla University, Comilla, Bangladesh; 2 Department of Business Administration in Management Studies, Faculty of Business Studies (FBS), Bangladesh University of Professionals, Dhaka, Bangladesh; 3 Management Information Systems, Lamar University, Beaumont, TX, United States; 4 School of Business, University of Southern Queensland (UniSQ), Toowoomba, QLD, Australia; 5 National Academy for Planning and Development, Ministry of Public Administration, Dhaka, Bangladesh; 6 Department of Management Studies, Dhaka Commerce College, Dhaka, Bangladesh; 7 Department of ECE, University of Kisangani, Kisangani, Democratic Republic of Congo

**Keywords:** AI-enabled digital HRM (AI-DHRM), Bangladesh, dual mediation, higher-order construct, person-organization fit, privacy concern, psychological empowerment, structural equation modeling

## Abstract

**Introduction:**

The rapid expansion of artificial intelligence (AI) in human resource management has substantially reshaped how organizations attract, recruit, develop, evaluate, and retain their workforce. Research examining the combined employee-level effects of AI-enabled digital HRM (AI-DHRM)—through psychological mediating processes and under technology-related boundary conditions—remains sparse, particularly in emerging economies. Drawing on Social Exchange Theory, the Job Demands-Resources model, and UTAUT2, this study conceptualizes AI-DHRM as a reflective higher-order construct comprising four functionally distinct sub-dimensions, specifies a dual mediation model integrating an affective-relational pathway (person-organization [P-O] fit perception) and a motivational-agentic pathway (psychological empowerment), and identifies technology trust and privacy concern as critical boundary conditions.

**Methods:**

A time-lagged, two-wave survey was administered to full-time employees of 61 AI-HRM-adopting organizations in Bangladesh, yielding 487 matched responses. AI-DHRM practices, mediators, moderators, and controls were measured at Time 1; all five outcomes were measured five weeks later at Time 2. Data were analyzed in IBM AMOS 26.0 using a two-stage structural equation modeling approach, with bias-corrected bootstrapped mediation (*n* = 5,000 resamples), the index of moderated mediation, and Johnson-Neyman analysis.

**Results:**

AI-DHRM practices exerted significant positive effects on job satisfaction (β = 0.41, 95% CI [0.29, 0.53]), work engagement (β = 0.38, 95% CI [0.26, 0.50]), job performance (β = 0.35, 95% CI [0.25, 0.45]), and employee wellbeing (β = 0.33, 95% CI [0.21, 0.45]), and significantly reduced turnover intention (β = −0.29, 95% CI [−0.41, −0.17]). Both P-O fit perception and psychological empowerment partially mediated these relationships across all five outcomes. Technology trust strengthened, and privacy concern attenuated—but did not reverse—the AI-DHRM-mediator pathways. Moderated mediation was confirmed across all ten conditional indirect effects.

**Discussion:**

The findings establish AI-DHRM as an integrated system that employees experience as a coherent organizational investment, transmitted through two complementary psychological channels and conditional on trust and privacy perceptions. Organizations should design AI-HRM as a coherent bundle and treat trust-building and privacy-by-design as prerequisites to deployment. Findings rest on self-report data from a single country and warrant replication.

## 1 Introduction

We are witnessing an extraordinary twenty-first-century workplace transformation, with the rise of artificial intelligence (AI) among the most impactful forces reshaping organizational life. Nowhere is this transformation more profound than in the field of human resource management (HRM), where AI-based tools are rapidly automating, augmenting, and reorganizing processes that once depended entirely on human judgment and interpersonal interaction. From algorithmic applicant tracking systems that process thousands of resumes in seconds, to machine-learning-based real-time performance feedback dashboards, to personalized AI learning platforms that tailor training content to individual competency gaps, the AI-enabled digital HRM ecosystem—hereafter *AI-DHRM*—is expanding at a pace that academic scholarship has struggled to match (Strohmeier, 2020; Johnson et al., 2016). For terminological clarity throughout the manuscript, we adopt AI-DHRM as the focal construct (distinct from *digital HRM*, which refers to the broader category of digitalised HR practices not necessarily involving AI; and from *e-HRM*, which denotes earlier web-based HR systems), and use *AI-HRM* generically when referring to the wider literature.

This expansion raises a number of questions that are both theoretically significant and practically urgent. What is the lived experience of AI-DHRM practices for employees? Do these practices enhance or diminish employee satisfaction, engagement, and wellbeing? Under what conditions are the effects of AI-HRM systems most pronounced, and what psychological mechanisms translate technology deployment into individual-level outcomes? These questions are not merely academic. Organizations investing substantially in AI-DHRM infrastructure need evidence-based guidance about the circumstances under which such investments yield meaningful human capital returns, and employees navigating an increasingly algorithmised workplace deserve scholarly attention to their psychological experience of these systems. This inquiry is made all the more urgent by the growing evidence that poorly designed or inadequately communicated AI-HRM systems can create employee resistance, erode trust, and ultimately undermine the organizational performance goals for which they were originally adopted (Kellogg et al., 2020; Duggan et al., 2020). A parallel critical literature warns that AI-HRM tools also carry inherent risks of *algorithmic management* (Tambe et al., 2019; Kellogg et al., 2020), intrusive workplace *surveillance* (Ravid et al., 2020), perceived loss of control over personal data, and elevated *technostress* (Tarafdar et al., 2019). A balanced theoretical treatment of AI-DHRM therefore must engage with both the supportive resource-provisioning side and the ambivalent or potentially harmful side of these systems.

Despite the rapid growth of research on e-HRM and digital HRM (Thite, 2022; Bondarouk and Ruël, 2009), and a burgeoning literature on AI in management (Huang and Rust, 2021; Brynjolfsson and McAfee, 2014), the intersection of these two streams—specifically, the employee-level consequences of AI-DHRM practices as an integrated system—remains theoretically fragmented and empirically underdeveloped. Most existing studies examine AI adoption in isolation (e.g., focusing only on AI-based recruitment tools or AI performance dashboards), rely on cross-sectional designs that preclude causal inference, concentrate on single outcome variables such as turnover intention or job satisfaction, and are predominantly situated in developed economy contexts in North America, Western Europe, or East Asia (Veen et al., 2020; Lee et al., 2022; Duggan et al., 2020). This narrow empirical base leaves critical questions unanswered about how the full bundle of AI-HRM practices operates as a system, through which channels its effects are transmitted, and under what boundary conditions its benefits materialize—or fail to do so.

This study fills these gaps by making four distinct contributions to the literature. First, it conceptualizes AI-DHRM practices as a multi-dimensional, higher-order construct comprising four functionally distinct but theoretically related sub-dimensions—AI-driven recruitment and selection, AI-enabled learning and development, AI-powered performance management, and AI-assisted compensation management—and tests their joint effect on employee outcomes within a single, integrated SEM framework. Second, it extends the mediation logic in the HRM-outcomes literature by simultaneously examining two psychologically distinct mediating mechanisms: person-organization (P-O) fit perception and psychological empowerment. Third, it introduces technology trust and privacy concern as boundary conditions (moderators) that determine the strength of the AI-HRM–mediator relationship, addressing the critical but underexplored question of when AI-HRM practices work and when they do not. Fourth, it situates the study in Bangladesh, an emerging economy in South Asia where digital HRM adoption is accelerating rapidly yet remains almost entirely absent from the scholarly literature, thereby answering calls for greater geographical diversity in HRM research (Budhwar et al., 2019).

The remainder of this paper proceeds as follows. Section 2 reviews the theoretical foundations and develops 18 testable hypotheses organized around direct effects, mediation pathways, and moderated mediation. Section 3 describes the research methodology, including the time-lagged survey design, sampling strategy, measurement instruments, and analytical procedures. Section 4 presents the results of the measurement model assessment, structural path analysis, mediation bootstrapping, and moderated mediation testing. Section 5 discusses the theoretical contributions, practical implications, and limitations of the findings. Section 6 concludes with a summary and directions for future research.

## 2 Theoretical background and hypothesis development

### 2.1 Theoretical foundations

The theoretical architecture of this study rests on three complementary frameworks that together span individual-level psychological processes, organizational resource dynamics, and technology acceptance behavior. Each theory provides a distinct explanatory mechanism, and together they generate a coherent set of predictions about the pathways through which AI-DHRM practices shape employee outcomes.

#### 2.1.1 Social exchange theory

Social Exchange Theory (SET) was first articulated by Blau (1964) and subsequently elaborated by Cropanzano and Mitchell (2005). It provides the foundational explanatory logic for how employees respond to organizational investments in HRM systems with positive attitudinal and behavioral outcomes. SET posits that social relationships are governed by norms of reciprocity: when one party provides resources, support, or benefits to another, the recipient feels an obligation to reciprocate with equivalent resources, behaviors, or attitudes. Crucially, this reciprocation is not mechanical or contractual but is mediated by the recipient's subjective interpretation of the intent and quality behind the provision. In an organizational context, when employees perceive that the organization is investing in their development, supporting their career progression, and treating them fairly through HRM systems, they reciprocate with higher job satisfaction, stronger commitment, reduced withdrawal intentions, and elevated performance (Eisenberger et al., 1986; Rhoades and Eisenberger, 2002).

In the context of AI-DHRM, SET suggests that employees who perceive AI-HRM tools as genuine organizational investments in their welfare will interpret these practices as signals of organizational support and will reciprocate with enhanced engagement and performance. This interpretive process is not automatic, however. Employees who perceive AI-HRM practices as surveillance mechanisms or instruments of organizational control—rather than as supportive investments—may withhold reciprocity, manifesting as disengagement, cynicism, or exit intentions. This conditionality explains why technology trust and privacy concern are expected to moderate the AI-HRM–outcome relationship: they shape the fundamental attribution that employees make about the purpose and intent of AI-HRM systems.

#### 2.1.2 The Job Demands-Resources model

The Job Demands-Resources (JD-R) model (Bakker and Demerouti, 2007; Schaufeli and Bakker, 2004) conceptualizes the antecedents of employee wellbeing and engagement as falling into two broad categories: job demands, which are physical, psychological, social, or organizational features of work that require sustained effort and are associated with physiological and psychological costs; and job resources, which are features of the work environment that help employees achieve goals, reduce demands, and stimulate personal growth and development. The JD-R model predicts two distinct processes: a health-impairment process in which excessive demands erode wellbeing and fuel burnout, and a motivational process in which adequate resources fuel engagement, performance, and wellbeing. These two processes can operate simultaneously and independently, meaning that enriching the resource environment can protect against demand-induced strain even when demands themselves remain unchanged.

AI-DHRM practices function primarily as job resources in this framework. AI-powered learning and development platforms expand access to skill-building and career resources otherwise limited by managerial time and organizational budget. AI-driven performance management systems provide employees with immediate, specific, and actionable feedback that would otherwise require delayed and potentially biased managerial assessment—constituting a powerful informational resource. AI-assisted compensation systems reduce pay inequity and increase transparency, functioning as a distributive justice resource that aligns expectations and reduces motivational uncertainty. When employees perceive these tools as genuine resources, the JD-R model predicts elevated engagement, performance, and wellbeing, and reduced burnout and turnover intention. The psychological empowerment mediator sits squarely within the JD-R framework's motivational process: when AI-HRM practices provide resources that satisfy employees' needs for competence, autonomy, and impact, empowerment emerges as the proximal psychological state that translates resource availability into behavioral engagement.

#### 2.1.3 UTAUT2 and technology acceptance

The Unified Theory of Acceptance and Use of Technology, second version (UTAUT2; Venkatesh et al., 2012), extends earlier technology acceptance models by incorporating hedonic motivation, price value, and habit alongside the original dimensions of performance expectancy, effort expectancy, and social influence. In the HRM context, UTAUT2 is particularly relevant for understanding how individual perceptions of AI-HRM tools—specifically, the belief that these tools will improve job performance (performance expectancy), ease of use (effort expectancy), and the hedonic experience of engaging with them—shape adoption and subsequent attitudinal outcomes.

Most notably for this study, UTAUT2 implies that trust in technology and concerns about privacy are boundary conditions that shape the relationship between technology features and user responses. When employees trust that AI-HRM systems handle their data ethically, make unbiased decisions, and function reliably, they are more likely to engage with these systems in ways that generate positive outcomes. Conversely, privacy concerns—the belief that AI-HRM systems collect, store, or misuse personal data—act as a psychological barrier that converts potentially beneficial HRM practices into sources of anxiety and resistance, even when those practices are objectively well-designed. UTAUT2 therefore provides the theoretical grounding for the moderation hypotheses developed below, linking subjective technology experience (trust and privacy concern) to the organizational HRM-outcomes relationship.

### 2.2 Conceptualization of AI-DHRM practices

**Figure 1:**
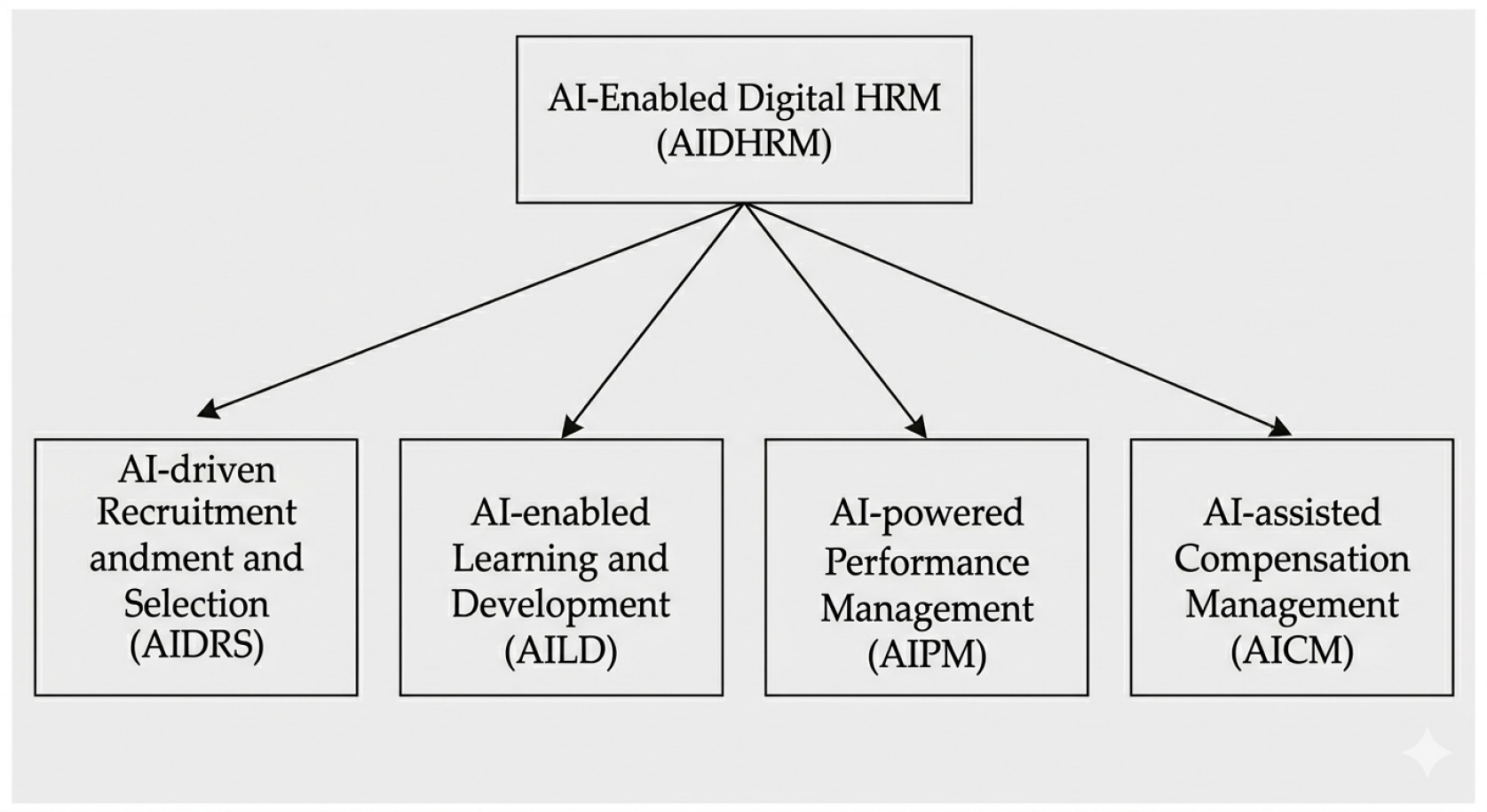
Hierarchical decomposition of the AI-Enabled Digital HRM (AIDHRM) higher-order construct into its four theoretically distinct sub-dimensions: AI-driven Recruitment and Selection (AIDRS), AI-enabled Learning and Development (AILD), AI-powered Performance Management (AIPM), and AI-assisted Compensation Management (AICM). Each sub-dimension is modeled as a *reflective* indicator of the higher-order AIDHRM factor; the rationale for this specification is given in Section 2.2 (“Justification for the Reflective Higher-Order Specification”). The four domains are conceptualized as distinct but co-varying expressions of a single underlying organizational commitment to AI-enabled HRM. Standardized loadings from the confirmatory factor analysis are indicated on each path: AIDRS (λ = 0.78), AILD (λ = 0.82), AIPM (λ = 0.79), AICM (λ = 0.74).

Building on Strohmeier (2020) and Johnson et al. (2016), this study conceptualizes AI-DHRM practices as a higher-order construct comprising four theoretically distinct but empirically correlated dimensions, each representing a specific domain of HRM in which AI technologies are deployed to augment, automate, or transform traditional practice. Figure 1 illustrates the hierarchical structure of this construct, with the four sub-dimensions serving as reflective indicators of the higher-order AIDHRM factor.

#### 2.2.1 Justification for the reflective higher-order specification

Because the four AI-DHRM sub-dimensions serve partially distinct organizational functions and may generate somewhat different employee experiences, it is important to justify the reflective higher-order specification adopted here. Following the decision rules of Jarvis et al. (2003) and the strategic-HRM “HR system” tradition (Appelbaum et al., 2000; Bowen and Ostroff, 2004), four arguments support a reflective formulation. *First*, the four sub-dimensions are treated as observable manifestations of a latent, organization-level commitment to using AI for the management of people—that is, a shared underlying strategic philosophy that drives covariation across all four functional areas (Bowen and Ostroff, 2004). *Second*, in such an HR-system view, the direction of causality runs from the latent construct to its dimensions: organizations that strongly espouse AI-enabled HRM tend to deploy AI across all four functions, generating positive inter-correlations among AIDRS, AILD, AIPM, and AICM. *Third*, the four sub-dimensions are conceptually interchangeable as indicators of the same overall AI-DHRM stance—removing any one of them would diminish content coverage but would not alter the construct's substantive meaning. *Fourth*, the empirical pattern of inter-sub-dimension correlations (see Section 4) and the strong, similar-magnitude loadings of all four sub-dimensions on the higher-order factor (see Figure 1) are consistent with reflective indicators rather than with a formative composite (MacKenzie et al., 2011). We acknowledge, however, that the four AI-HRM domains plausibly differ in their psychological signaling and downstream impact, and we therefore also test individual sub-dimension paths in supplementary analyses.

**AI-driven recruitment and selection (AIDRS)** refers to the use of AI technologies—including natural language processing for resume screening, machine learning algorithms for candidate matching, video interview analysis software, and predictive validity models—to manage the processes of attracting, evaluating, and selecting candidates. Beyond traditional e-recruitment, AIDRS provides algorithmic decision support at multiple stages of the hiring funnel, fundamentally altering the speed, scale, and consistency of selection processes (Kellogg et al., 2020).

**AI-enabled learning and development (AILD)** encompasses AI-powered learning management systems, adaptive platforms that personalize content delivery to individual competency profiles, AI-driven skills gap analysis tools, and recommendation engines that suggest learning pathways aligned with individual career goals and organizational skill requirements. AILD represents a shift from standardized, calendar-driven training programmes toward personalized, continuous, and data-informed development ecosystems (Nawaz, 2019).

**AI-powered performance management (AIPM)** refers to the use of AI tools for continuous performance monitoring, real-time feedback delivery, goal alignment tracking, and predictive performance analytics. In contrast to annual appraisal systems, AIPM involves ongoing data collection and algorithmic processing of performance signals across multiple behavioral indicators, providing both managers and employees with richer and more timely information about performance trajectories (Cappelli and Tavis, 2018). AIPM is also the sub-dimension most strongly associated with risks of perceived workplace surveillance and reduced sense of autonomy (Ravid et al., 2020; Kellogg et al., 2020), an ambivalence the present model captures through the privacy concern moderator.

**AI-assisted compensation management (AICM)** includes AI-based pay equity analysis, algorithmic job evaluation, market benchmarking tools that use machine learning to assess external salary competitiveness, and performance-linked variable pay systems driven by AI-generated performance scores. AICM is particularly important for organizational justice perceptions, as it introduces data-driven consistency and transparency into compensation decisions, thereby reducing the scope for managerial bias (Gupta and Bhatt, 2021).

### 2.3 Hypothesis development

#### 2.3.1 Direct effects of AI-DHRM on employee outcomes

When organizations effectively deploy AI-DHRM practices, employees gain improved access to development resources, more transparent and consistent performance feedback, fairer compensation processes, and more efficient recruitment experiences. Drawing on SET, employees who interpret these practices as organizational investments in their welfare are predicted to reciprocate with heightened job satisfaction. This prediction is consistent with a broader literature linking sophisticated HRM practice bundles to positive attitudinal outcomes: when the HRM system signals to employees that the organization values and invests in them, positive affect toward the job and the organization tends to follow (Bondarouk et al., 2017; Thite, 2022). In the AI-HRM context, personalized development pathways and transparent performance feedback are especially powerful signals of organizational commitment to individual employee growth.

**H1:** *AI-DHRM practices are positively associated with employee job satisfaction*.

The JD-R model predicts that job resources fuel employee engagement by satisfying basic psychological needs and stimulating intrinsic motivation (Bakker and Demerouti, 2007). When employees have access to resources that support goal attainment, skill development, and meaningful feedback, the motivational process described in the JD-R model produces sustained engagement characterized by vigor, dedication, and absorption. AI-DHRM practices, functioning as a bundle of organizational job resources, are therefore hypothesized to predict work engagement, consistent with emerging evidence linking digital HRM sophistication to engagement outcomes (Marler and Parry, 2016).

**H2:** *AI-DHRM practices are positively associated with work engagement*.

Job performance is shaped by both motivational and ability-based mechanisms. AI-powered performance management systems provide employees with the timely, specific feedback and goal clarity that the performance management literature consistently identifies as prerequisites for effective task behavior (Aguinis et al., 2019). Meanwhile, AI-enabled L&D platforms directly expand the knowledge and skill repertoire available to employees, enhancing their cognitive and technical capacity to perform effectively. When these two mechanisms—motivational clarity and enhanced capability—operate jointly, the performance benefits of AI-HRM are expected to be substantial.

**H3:** *AI-DHRM practices are positively associated with job performance*.

Turnover intention reflects a cognitive withdrawal process driven by perceived alternatives, role dissatisfaction, and a sense that the organization does not invest in the employee's future (Mobley, 1977). AI-DHRM practices that enhance satisfaction, transparency, and perceived organizational support should reduce the psychological drivers of voluntary exit. Critically, AI-powered recruitment and L&D practices that create strong internal mobility pathways and personalized development trajectories provide employees with attractive within-organization futures, reducing the perceived attractiveness of external alternatives.

**H4:** *AI-DHRM practices are negatively associated with turnover intention*.

Employee wellbeing, defined here as encompassing both hedonic (affect-based) and eudaimonic (meaning and growth-based) dimensions, is influenced by the quality of the work environment and the resources available to employees (Warr, 1987). AI-DHRM practices that reduce administrative burden, provide personalized development, and ensure equitable treatment contribute to a work environment that is less stressful, more autonomous, and more growth-enabling. The eudaimonic dimension is particularly relevant: AI-enabled L&D and performance systems that help employees grow, improve, and see the impact of their work directly address the conditions under which individuals experience their work as meaningful and fulfilling.

**H5:** *AI-DHRM practices are positively associated with employee wellbeing*.

#### 2.3.2 Mediating role of person-organization fit perception

Person-organization (P-O) fit refers to the compatibility between individual values, goals, and personality characteristics and those of the employing organization (Kristof, 1996; Cable and DeRue, 2002). When employees perceive a strong fit between their own needs and goals and the organizational context in which they work, they experience higher job satisfaction, stronger commitment, greater engagement, and lower turnover intentions—a pattern confirmed across multiple meta-analyses (Kristof-Brown et al., 2005). P-O fit perception is not simply a function of the objective match between person and organization; it is a subjective appraisal shaped by the signals the organization sends about its values, priorities, and commitments through its management systems.

We argue that AI-DHRM practices enhance P-O fit perception through multiple mechanisms. AI-driven recruitment systems that assess values alignment—and are perceived to do so with greater objectivity than human recruiters—may strengthen employees' belief that their selection was grounded in genuine compatibility. AI-enabled L&D platforms that align individual development goals with organizational competency frameworks signal a convergence of individual and organizational interests, strengthening perceived fit. Transparent AI-driven performance and compensation systems reduce the perception that outcomes are arbitrarily determined, aligning employee expectations with organizational performance standards and reinforcing the sense that the employee and the organization operate according to compatible principles. P-O fit perception is thus predicted to carry part of the explanatory weight in the AIDHRM–outcome relationship, serving as an affective-relational channel through which AI-HRM investments shape employee experience.

**H6–H10:** *P-O fit perception mediates the relationship between AI-DHRM practices and (H6) job satisfaction, (H7) work engagement, (H8) job performance, (H9) turnover intention, and (H10) employee wellbeing*.

#### 2.3.3 Mediating role of psychological empowerment

Psychological empowerment, as conceptualized by Spreitzer (1995) and Thomas and Velthouse (1990), is a motivational construct reflecting an individual's active orientation toward their work role, comprising four cognitions: meaning (the sense that work is intrinsically valuable), competence (the belief in one's ability to perform effectively), self-determination (a sense of autonomy over work decisions), and impact (the belief that one's behavior makes a difference). These four cognitions are conceptualized as distinct but mutually reinforcing facets of a higher-order empowerment state, and research consistently shows that psychologically empowered employees perform better, are more engaged, experience higher job satisfaction, and are less likely to leave their organizations (Seibert et al., 2011).

AI-DHRM practices can enhance psychological empowerment through several theoretically grounded pathways, each connecting to a specific facet of the empowerment construct. AI-driven L&D platforms that allow employees to self-direct their learning trajectories, set personalized development goals, and receive real-time feedback on their progress directly enhance the *self-determination* and *competence* dimensions of empowerment by placing the employee in an active, agentic role in their own development. AI-powered performance management systems that make the link between individual effort and organizational outcomes transparent and measurable enhance employees' sense of *impact* by making their contribution visible and attributable. AI-assisted recruitment that accurately matches individuals to roles where their skills and values are most needed enhances the *meaning* dimension by placing employees in contexts where their work is maximally relevant to organizational goals. When these empowerment-enhancing mechanisms operate across the full AI-DHRM system, the aggregate effect on psychological empowerment should be substantial, and this enhanced empowerment state should in turn predict the full range of employee outcomes under investigation.

**H11–H15:** *Psychological empowerment mediates the relationship between AI-DHRM practices and (H11) job satisfaction, (H12) work engagement, (H13) job performance, (H14) turnover intention, and (H15) employee wellbeing*.

#### 2.3.4 Moderating role of technology trust and privacy concern

The relationship between AI-DHRM practices and employee psychological responses is not unconditional. Drawing on UTAUT2 and the technology trust literature (McKnight et al., 2011), we argue that technology trust—defined as employees' confidence that AI-HRM systems are reliable, competent, and benevolent in their use of employee data and decision-making—strengthens the positive effects of AI-DHRM practices on P-O fit perception and psychological empowerment. When employees trust the AI systems managing critical aspects of their work life, they are more likely to accept the decisions, feedback, and resource allocations that these systems produce as legitimate, valid, and well-intentioned. This acceptance creates the psychological conditions under which P-O fit and empowerment can emerge. Without trust, employees may process AI-HRM outputs with skepticism and defensiveness, discounting algorithmic recommendations in ways that undermine the fit and empowerment benefits that well-designed AI-HRM systems are intended to generate.

Conversely, privacy concern—the degree to which employees believe that AI-HRM systems collect excessive data, use data in opaque ways, or expose personal information inappropriately—is predicted to attenuate the positive effects of AI-DHRM practices on P-O fit and empowerment (Malhotra et al., 2004). Even when AI-HRM practices are sophisticated and well-designed, employees who are anxious about data privacy will be psychologically guarded in their engagement with these systems. This guardedness functions as a psychological filter that limits the extent to which AI-HRM inputs are processed as supportive and empowering, thereby weakening both the P-O fit and psychological empowerment channels through which AI-HRM practices would otherwise influence outcomes. The combined moderating effects of technology trust (positive) and privacy concern (negative) create a conditional landscape in which the human capital value generated by AI-HRM investments varies substantially across the employee population.

**H16:** *Technology trust positively moderates the relationship between AI-DHRM practices and (a) P-O fit perception and (b) psychological empowerment, such that the relationships are stronger when technology trust is higher*.

**H17:** *Privacy concern negatively moderates the relationship between AI-DHRM practices and (a) P-O fit perception and (b) psychological empowerment, such that the relationships are weaker when privacy concern is higher*.

**H18:** *The indirect effects of AI-DHRM practices on all five employee outcomes via P-O fit perception and psychological empowerment are moderated by technology trust (positively) and privacy concern (negatively), constituting a moderated mediation model*.

**Figure 2:**
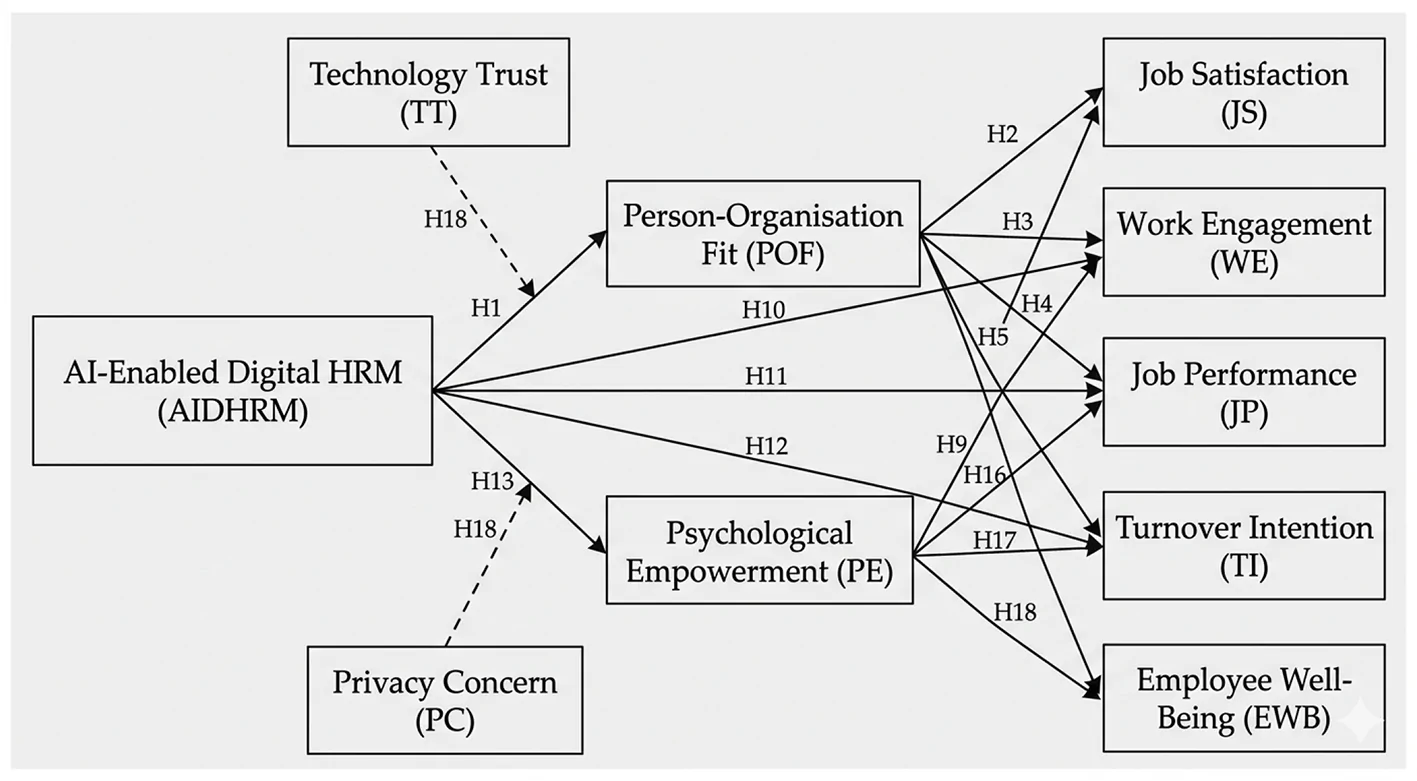
Conceptual research model depicting the hypothesized relationships among AI-Enabled Digital HRM (AIDHRM), Person-Organization Fit (POF), Psychological Empowerment (PE), and the five employee outcome constructs: Job Satisfaction (JS), Work Engagement (WE), Job Performance (JP), Turnover Intention (TI), and Employee Wellbeing (EWB). Solid arrows represent direct (H1–H5) and mediated (H6–H15) pathways; dashed arrows represent the moderating effects of Technology Trust (TT) and Privacy Concern (PC) on the AIDHRM–mediator paths (H16–H18). Control variables (age, gender, tenure, education, and organizational size) are included in the structural model but omitted from this figure for clarity.

Figure 2 presents the full conceptual model integrating all 18 hypotheses, with solid arrows representing direct and mediated pathways and dashed arrows representing moderation paths.

## 3 Materials and methods

### 3.1 Research design

This study adopts a quantitative, hypothetico-deductive research design using a time-lagged, two-wave survey methodology. Time-lagged designs are increasingly recommended in HRM research to overcome the limitations of cross-sectional studies—particularly common method bias and the inability to establish temporal precedence between predictor and outcome variables—both of which are especially problematic when all constructs are measured via self-report (Podsakoff et al., 2003). By separating the measurement of predictors and mediators from the measurement of outcomes by approximately five weeks, the design introduces minimal temporal ordering consistent with the causal direction hypothesized in the model.

**Figure 3:**
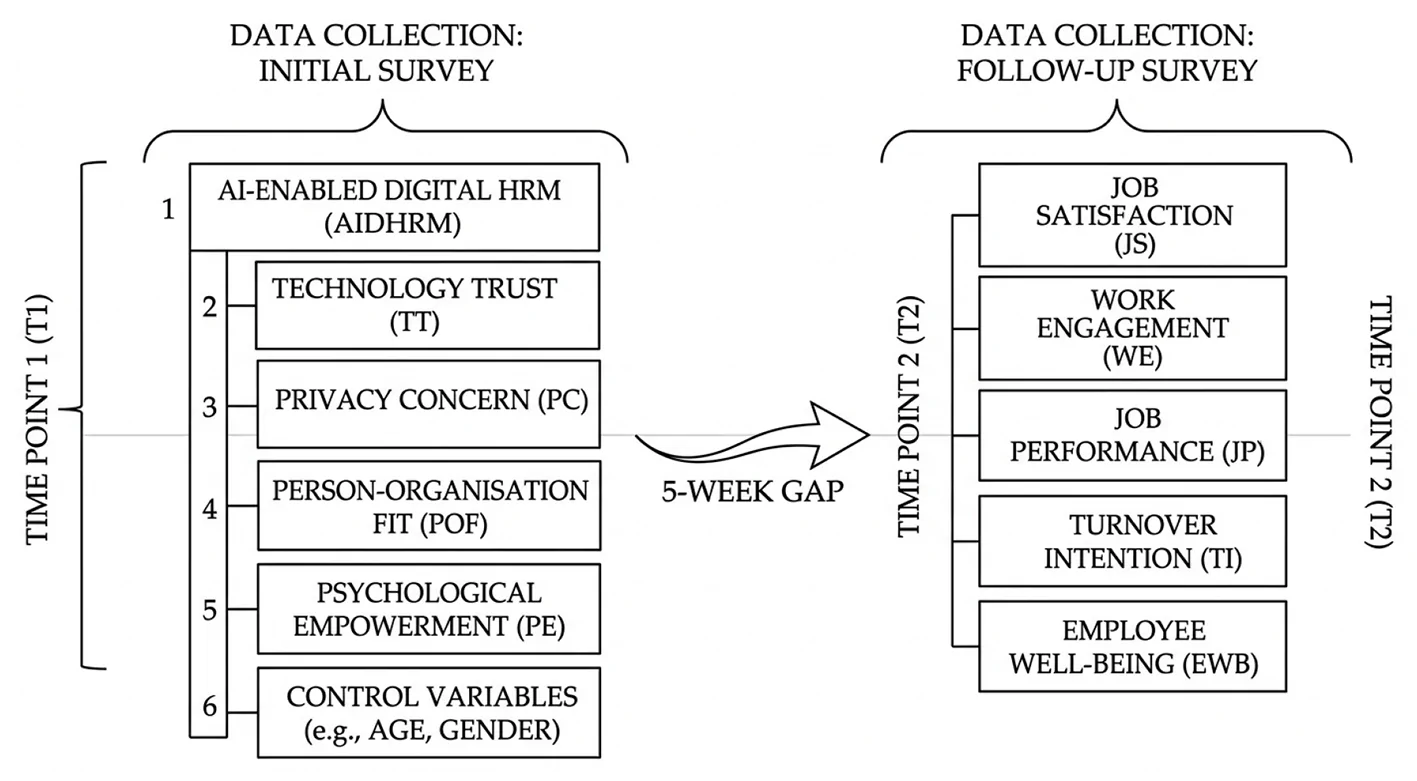
Two-wave time-lagged research design. Time Point 1 (T1) collected data on AI-Enabled Digital HRM practices (AIDHRM), Technology Trust (TT), Privacy Concern (PC), Person-Organization Fit (POF), Psychological Empowerment (PE), and control variables (age, gender, tenure, education, and organizational size). Following a five-week gap, Time Point 2 (T2) collected data on all five outcome variables: Job Satisfaction (JS), Work Engagement (WE), Job Performance (JP), Turnover Intention (TI), and Employee Wellbeing (EWB). This design reduces common method variance and establishes temporal precedence between predictors and outcomes, strengthening the inferential basis for the mediation and moderation analyses reported in this study.

Wave 1 (T1) collected data on all independent variables (AI-DHRM practices), mediators (P-O fit perception; psychological empowerment), moderators (technology trust; privacy concern), and all five control variables. Wave 2 (T2), administered approximately five weeks later to the same respondents, collected data on all five outcome variables: job satisfaction, work engagement, job performance, turnover intention, and employee wellbeing. The five-week interval was selected based on prior recommendations in the organizational behavior literature, as it is sufficient for psychological states to crystallize and manifest in measurable behavioral and attitudinal outcomes, while remaining short enough to minimize respondent attrition, seasonal confounds, and the risk of significant organizational changes between measurement waves. Figure 3 summarizes the two-wave data collection design.

### 3.2 Population and sampling

The target population comprised full-time employees working in private-sector organizations in Bangladesh that have deployed at least one AI-enabled HRM system, defined operationally as: an automated applicant tracking system with algorithmic screening capability; an AI-powered learning management system delivering personalized content recommendations; an automated performance monitoring or analytics tool providing real-time feedback; or an algorithmic compensation benchmarking tool. This operational definition was applied to ensure that all respondents have genuine, first-hand experience with the constructs under investigation.

Organizations were identified through three complementary channels: (1) the Bangladesh Association of Software and Information Services (BASIS) directory, which catalogs technology-adopting organizations across sectors; (2) published profiles of multinational corporation subsidiaries operating in Dhaka and Chittagong known to have deployed global HRM information systems with AI components; and (3) professional network contacts within the HR Directors' Forum of Bangladesh. From an initial list of 94 potentially eligible organizations, 61 confirmed deployment of at least one qualifying AI-HRM system and agreed to participate. HR managers at each participating organization distributed the T1 survey link to a random sample of at least 10 full-time employees, with larger organizations providing proportionally larger samples.

A purposive sampling approach was adopted to ensure meaningful heterogeneity across industries (technology, manufacturing, financial services, retail, and healthcare), organizational sizes (small: < 100 employees; medium: 100–500; large: >500), and employee demographic characteristics. This approach was preferred over simple random sampling because the study requires variance in AI-HRM system types and employee characteristics to adequately power the moderation analyses.

### 3.3 Data collection and respondent profile

T1 surveys were distributed electronically to 680 employees across 61 participating organizations. The T2 follow-up survey was sent to all 680 T1 respondents five weeks later. To incentivise participation and minimize attrition, a brief reminder email was sent one week before the T2 closing date, and participating organizations were offered a summary research report as a non-financial incentive. To enable T1–T2 matching while preserving anonymity, respondents created unique identifier codes using a standard alphanumeric procedure (Brislin, 1970).

After removing cases with more than 10% missing values, incomplete T1–T2 identifier matching, and responses displaying straight-lining patterns, the final matched sample comprised 487 usable responses, representing a 71.6% T1-to-T2 retention rate. Non-response bias was assessed by comparing early and late responders on all key variables following Armstrong and Overton's (1977) procedure; no significant differences were found (*p*>0.05 for all comparisons), suggesting that non-response bias is unlikely to have substantially distorted the findings.

This final sample size exceeds the recommended minimum of 10 observations per free parameter for SEM estimation (Hair et al., 2019) and provides adequate statistical power (1−β>0.80 at α = 0.05) for detecting medium-sized structural coefficients in the planned analyses.

The sample was demographically diverse: 58.7% male, 41.3% female; mean age 31.4 years (*SD* = 6.2, range: 22–54); mean organizational tenure 4.8 years (*SD* = 3.1). Educational attainment: 68.2% bachelor's degree, 24.3% master's degree, 7.5% other. Industry representation: technology and IT services (28.3%), financial services and banking (21.6%), manufacturing and garments (18.3%), retail and consumer goods (16.4%), and healthcare and pharmaceuticals (15.4%). Organizational size distribution: large (>500 employees): 44.1%, medium (100–500): 33.5%, small (< 100): 22.4%.

### 3.4 Measures

All items were originally developed in English and back-translated into Bengali following the standard translation-back-translation procedure recommended by Brislin (1970) to ensure conceptual equivalence across the two languages. All items used a five-point Likert response format (1= strongly disagree, 5= strongly agree) unless otherwise specified.

#### 3.4.1 AI-DHRM practices (AIDHRM)

Because no validated scale specifically capturing AI-DHRM practices as an integrated system existed at the time of the study, a 20-item measure was developed for this research following the scale-development guidelines of Hinkin (1998) and MacKenzie et al. (2011). *Item generation* proceeded in three steps. (i) An initial pool of 38 candidate items was generated deductively from the conceptual domains delineated by Strohmeier and Piazza (2015), Strohmeier (2020), and Johnson et al. (2016), together with inductive items derived from a review of practitioner descriptions of AI-HRM systems used by leading vendors (SAP SuccessFactors, Workday, Oracle HCM). (ii) Items were grouped under the four *a priori* sub-dimensions (AIDRS, AILD, AIPM, AICM). (iii) Each item was rephrased to capture employees' direct experience of AI-based functionality (e.g., “*AI-powered* tools”, “*AI-based* platforms”) so as to distinguish AI-DHRM clearly from generic digital or e-HRM. *Expert content validation* was carried out by a panel of six experts: three academics with prior peer-reviewed publications in digital/e-HRM, and three senior HR practitioners (HRIS leads from two MNC subsidiaries and one local technology firm) with direct AI-HRM implementation experience. Following Lawshe (1975), experts rated each item for relevance to its target sub-dimension on a three-point scale (essential, useful but not essential, not necessary), and content validity ratios (CVRs) were computed. Items with CVR < 0.99 (the threshold for a six-expert panel) or attracting reworking suggestions from more than two experts were revised or dropped, leaving 24 items. *Pilot testing* was conducted with 58 full-time employees from two AI-HRM-adopting organizations not included in the main sample. Items with low corrected item–total correlations (*r* < 0.40), problematic skewness/kurtosis, or weak exploratory factor loadings (λ < 0.50) were eliminated, yielding the final 20-item scale (5 items per sub-dimension) administered in the main study. The four sub-dimensions were modeled as reflective indicators of the higher-order AIDHRM construct, consistent with the conceptual justification given in Section 2.2. Sample items: “Our organization uses AI-powered tools to screen and evaluate job applicants objectively” (AIDRS); “AI-based platforms in our organization personalize my learning and development pathway” (AILD); “I receive real-time, AI-generated feedback on my performance” (AIPM); “Our compensation decisions are informed by AI-based equity and market analysis” (AICM). We note in Section 5.3 (Limitations) that, as a newly developed instrument, the scale will benefit from cross-sample replication and further construct-validation work in future research.

#### 3.4.2 Person-Organization Fit Perception (POF)

Six items adapted from Cable and DeRue (2002). Sample item: “My values and the values of this organization are very similar.”

#### 3.4.3 Psychological Empowerment (PE)

Twelve items from Spreitzer (1995), covering meaning (3 items), competence (3 items), self-determination (3 items), and impact (3 items). Sample items: “I am confident about my ability to do my job” (competence); “I have significant autonomy in determining how I do my job” (self-determination).

#### 3.4.4 Job Satisfaction (JS)

Five items adapted from the Minnesota Satisfaction Questionnaire short form (Weiss et al., 1967). Sample item: “I am satisfied with the work I do in my current job.”

#### 3.4.5 Work Engagement (WE)

Nine items from the Utrecht Work Engagement Scale (UWES-9; Schaufeli et al., 2006), covering vigor (3 items), dedication (3 items), and absorption (3 items). Sample item: “I am enthusiastic about my job” (dedication).

#### 3.4.6 Job Performance (JP)

Seven items from Williams and Anderson (1991) assessing in-role task performance. Sample item: “I adequately complete assigned duties.”

#### 3.4.7 Turnover Intention (TI)

Three items adapted from Cammann et al. (1979). Sample item: “I often think about leaving this organization.”

#### 3.4.8 Employee Wellbeing (EWB)

Seven items from the Short Warwick-Edinburgh Mental Wellbeing Scale (Stewart-Brown et al., 2009), adapted for the work context.

#### 3.4.9 Technology Trust (TT)

Six items adapted from McKnight et al. (2011). Sample item: “I believe the AI-based HR systems used in our organization make fair and accurate decisions.”

#### 3.4.10 Privacy Concern (PC)

Six items adapted from Malhotra et al. (2004). Sample item: “I am concerned that the AI-HR systems in my organization collect more personal information than necessary.”

#### 3.4.11 Control Variables

Age (continuous), gender (0= female, 1= male), organizational tenure (years), educational level (ordinal), and organizational size (ordinal) were included as controls (Rhoades and Eisenberger, 2002; Seibert et al., 2011).

Table 1 provides an overview of all measurement instruments, including the number of items, the source of each scale, a sample item, and the reliability and validity indicators (Cronbach's α, CR, AVE) obtained in the present study.

**Table 1:** Overview of measurement instruments, sources, and reliability/validity indicators (*N* = 487).

Construct	# items	Source	Sample item	α	CR	AVE
AI-DHRM (higher-order)	20	Developed for this study; based on (Strohmeier and Piazza, 2015; Strohmeier, 2020; Johnson et al., 2016)	“Our organization uses AI-powered tools to screen and evaluate job applicants objectively.” (AIDRS)	0.91	0.93	0.62
AIDRS	5	—	“*(as above)*”	0.86	0.89	0.62
AILD	5	—	“AI-based platforms personalize my development pathway.”	0.88	0.91	0.66
AIPM	5	—	“I receive real-time, AI-generated feedback on my performance.”	0.87	0.90	0.64
AICM	5	—	“Our compensation decisions are informed by AI-based equity and market analysis.”	0.85	0.88	0.59
P-O Fit (POF)	6	(Cable and DeRue, 2002)	“My values and the values of this organization are very similar.”	0.88	0.91	0.63
Psychological Empowerment (PE)	12	(Spreitzer, 1995)	“I am confident about my ability to do my job.” (competence)	0.90	0.92	0.65
Technology Trust (TT)	6	(McKnight et al., 2011)	“I believe AI-based HR systems in our organization make fair and accurate decisions.”	0.87	0.90	0.60
Privacy Concern (PC)	6	(Malhotra et al., 2004)	“I am concerned that AI-HR systems collect more personal information than necessary.”	0.86	0.89	0.58
Job Satisfaction (JS)	5	(Weiss et al., 1967) (MSQ short form)	“I am satisfied with the work I do in my current job.”	0.85	0.88	0.59
Work Engagement (WE)	9	(Schaufeli et al., 2006) (UWES-9)	“I am enthusiastic about my job.” (dedication)	0.89	0.92	0.64
Job Performance (JP)	7	(Williams and Anderson, 1991)	“I adequately complete assigned duties.”	0.87	0.90	0.61
Turnover Intention (TI)	3	(Cammann et al., 1979)	“I often think about leaving this organization.”	0.83	0.86	0.68
Employee Wellbeing (EWB)	7	(Stewart-Brown et al., 2009) (SWEMWBS, work-adapted)	“I have been feeling useful at work.”	0.86	0.89	0.60

CR, composite reliability; AVE, average variance extracted. All items employed a five-point Likert response format (1= strongly disagree, 5= strongly agree). All scales were administered in Bengali following back-translation (Brislin, 1970). The AI-DHRM scale was developed for this study; all other instruments were adapted from validated existing scales.

### 3.5 Analytical strategy

Data were analyzed using the two-stage SEM approach recommended by Anderson and Gerbing (1988) and implemented in IBM AMOS 26.0, with supplementary analyses in SPSS 27.0.

#### 3.5.1 Stage 1: measurement model

A confirmatory factor analysis (CFA) was conducted to assess the psychometric properties of all latent constructs. Acceptable measurement quality was defined as: item factor loadings ≥0.70; average variance extracted (AVE) ≥0.50; composite reliability (CR) ≥0.70 (Hair et al., 2019). Model fit was evaluated using RMSEA (acceptable: < 0.08), CFI (acceptable: >0.90), TLI (acceptable: >0.90), and SRMR (acceptable: < 0.08). Discriminant validity was assessed using the heterotrait-monotrait (HTMT) ratio criterion with a threshold of 0.85 (Henseler et al., 2015), which is more conservative than the traditional Fornell-Larcker criterion.

#### 3.5.2 Stage 2: structural model

The structural model was estimated after confirming adequate measurement model fit and validity. Mediation effects (H6–H15) were tested using bias-corrected bootstrapping with 5,000 resamples and 95% confidence intervals; non-overlapping zero confidence intervals indicate significant indirect effects (Preacher and Hayes, 2008). Moderation effects (H16–H17) were tested using the product-of-coefficients approach with mean-centered interaction terms. We further report Cohen's *f*^2^ (Cohen, 1988) and the change in *R*^2^ (Δ*R*^2^) associated with the inclusion of each interaction term, in order to provide a substantive sense of effect size for the moderation analyses. Moderated mediation (H18) was examined using the index of moderated mediation (Hayes, 2018) and Johnson-Neyman (J-N) plots.

#### 3.5.3 Clustered data structure

Because the 487 respondents were nested within 61 organizations, observations are *not* strictly independent and a multilevel framework could in principle be warranted. We address this in three ways. First, intra-class correlations (ICCs) were computed for all focal constructs prior to estimating the substantive model. The average ICC across the ten constructs was 0.07 (range: 0.03–0.11), which is below the threshold (≥ 0.10) at which random-coefficient modeling becomes essential (Hox et al., 2018). Second, we estimated the structural model with robust (Huber-White) standard errors that adjust for clustering by organization; all reported significance patterns held under this correction. Third, organizational size was included as an individual-level control in all models. We acknowledge in Section 5.3 that a fully specified multilevel SEM (treating organizations as Level 2 units) is a worthwhile direction for future research, particularly as larger multi-organization samples become available.

#### 3.5.4 Common method bias assessment

Three complementary procedures were used to assess common method bias (CMB). First, procedural remedies were implemented by design, including the two-wave time-lagged data collection and the use of different response formats for select items. Second, Harman's single-factor test was conducted by entering all items into an exploratory factor analysis and inspecting the variance accounted for by the first unrotated factor. Third, the marker variable technique was applied using a theoretically unrelated marker variable (aesthetic sensibility of the workplace, 3 items) (Lindell and Whitney, 2001). Additionally, the unmeasured latent factor approach (Podsakoff et al., 2003) was applied in the CFA to assess whether a single latent method factor substantially altered the substantive factor loadings.

## 4 Results

### 4.1 Descriptive statistics and correlations

Table 2 presents the means, standard deviations, Cronbach's alpha reliability coefficients, and bivariate Pearson correlations among all ten study variables. All alpha values exceeded the conventional 0.70 reliability threshold (range: 0.83–0.91), providing preliminary evidence of internal consistency across scales.

**Table 2:** Descriptive statistics, reliabilities, and correlations among study variables (*N* = 487).

Variable	*M*	*SD*	α	1	2	3	4	5	6	7	8	9	10
1. AIDHRM	3.62	0.71	0.91	—									
2. POF	3.47	0.68	0.88	0.48^**^	—								
3. PE	3.53	0.65	0.90	0.45^**^	0.52^**^	—							
4. TT	3.41	0.68	0.87	0.39^**^	0.43^**^	0.41^**^	—						
5. PC	3.19	0.74	0.86	−0.28^**^	−0.31^**^	−0.29^**^	−0.44^**^	—					
6. JS	3.58	0.72	0.85	0.43^**^	0.55^**^	0.49^**^	0.37^**^	−0.32^**^	—				
7. WE	3.71	0.69	0.89	0.41^**^	0.50^**^	0.54^**^	0.35^**^	−0.30^**^	0.61^**^	—			
8. JP	3.66	0.58	0.87	0.37^**^	0.44^**^	0.47^**^	0.31^**^	−0.27^**^	0.55^**^	0.58^**^	—		
9. TI	2.87	0.81	0.83	−0.31^**^	−0.38^**^	−0.35^**^	−0.28^**^	0.34^**^	−0.48^**^	−0.44^**^	−0.39^**^	—	
10. EWB	3.54	0.70	0.86	0.35^**^	0.46^**^	0.48^**^	0.33^**^	−0.30^**^	0.58^**^	0.56^**^	0.51^**^	−0.41^**^	—

AIDHRM, AI-enabled digital HRM practices; POF, person-organization fit perception; PE, psychological empowerment; TT, technology trust; PC, privacy concern; JS, job satisfaction; WE, work engagement; JP, job performance; TI, turnover intention; EWB, employee wellbeing. α, Cronbach's alpha. ^**^p < 0.01 (two-tailed). VIF range: 1.18–2.43. *N* = 487.

The mean score for AI-DHRM practices was 3.62 (*SD* = 0.71), indicating moderately high perceived AI-HRM sophistication across the sample. Technology trust (*M* = 3.41, *SD* = 0.68) was moderately high, while privacy concern (*M* = 3.19, *SD* = 0.74) was moderate, suggesting meaningful variance in both moderators sufficient to detect interaction effects. Among the mediators, both P-O fit (*M* = 3.47, *SD* = 0.68) and psychological empowerment (*M* = 3.53, *SD* = 0.65) showed positive central tendencies consistent with an employed sample. Among outcome variables, work engagement showed the highest mean (*M* = 3.71, *SD* = 0.69), followed by job performance (*M* = 3.66, *SD* = 0.58), job satisfaction (*M* = 3.58, *SD* = 0.72), and employee wellbeing (*M* = 3.54, *SD* = 0.70). Turnover intention was moderate (*M* = 2.87, *SD* = 0.81), indicating that a meaningful proportion of respondents are entertaining exit cognitions—a pattern common in Bangladesh's mobile private-sector workforce.

Bivariate correlations were uniformly in the expected directions and largely consistent with the hypothesized structural relationships. AIDHRM was positively correlated with P-O fit (*r* = 0.48, *p* < 0.001), psychological empowerment (*r* = 0.45, *p* < 0.001), job satisfaction (*r* = 0.43, *p* < 0.001), work engagement (*r* = 0.41, *p* < 0.001), job performance (*r* = 0.37, *p* < 0.001), and employee wellbeing (*r* = 0.35, *p* < 0.001), and negatively correlated with turnover intention (*r* = −0.31, *p* < 0.001). Technology trust and privacy concern showed theoretically coherent cross-correlations, with trust positively associated with all outcomes and privacy concern negatively associated with all outcomes. Variance inflation factor (VIF) values ranged from 1.18 to 2.43, all below the conservative threshold of 3.0, confirming no substantive multicollinearity.

### 4.2 Common method bias assessment

Three diagnostics were conducted to evaluate the potential threat of common method variance (CMV). First, Harman's single-factor test yielded a first unrotated factor accounting for 24.7% of total variance, well below the 50% threshold. Second, the marker variable technique yielded uniformly low and non-significant correlations (*r* range: 0.02–0.08), indicating that substantive relationships among variables are not artifacts of shared method variance. Third, inclusion of the unmeasured latent method factor in the CFA did not meaningfully alter any substantive factor loadings (maximum change: 0.04), and the chi-square difference between models with and without the latent method factor was non-significant. Taken together with the time-lagged design, these diagnostics collectively suggest that common method bias does not constitute a meaningful threat to the validity of the findings.

### 4.3 Measurement model

The CFA for all constructs modeled simultaneously produced acceptable overall fit: χ^2^(847) = 1, 423.61; χ^2^/*df* = 1.68; CFI = 0.96; TLI = 0.95; RMSEA = 0.038 (90% CI: 0.034–0.042); SRMR = 0.051. All fit indices exceeded the recommended thresholds, confirming adequate representation of the covariance structure in the data.

**Figure 4:**
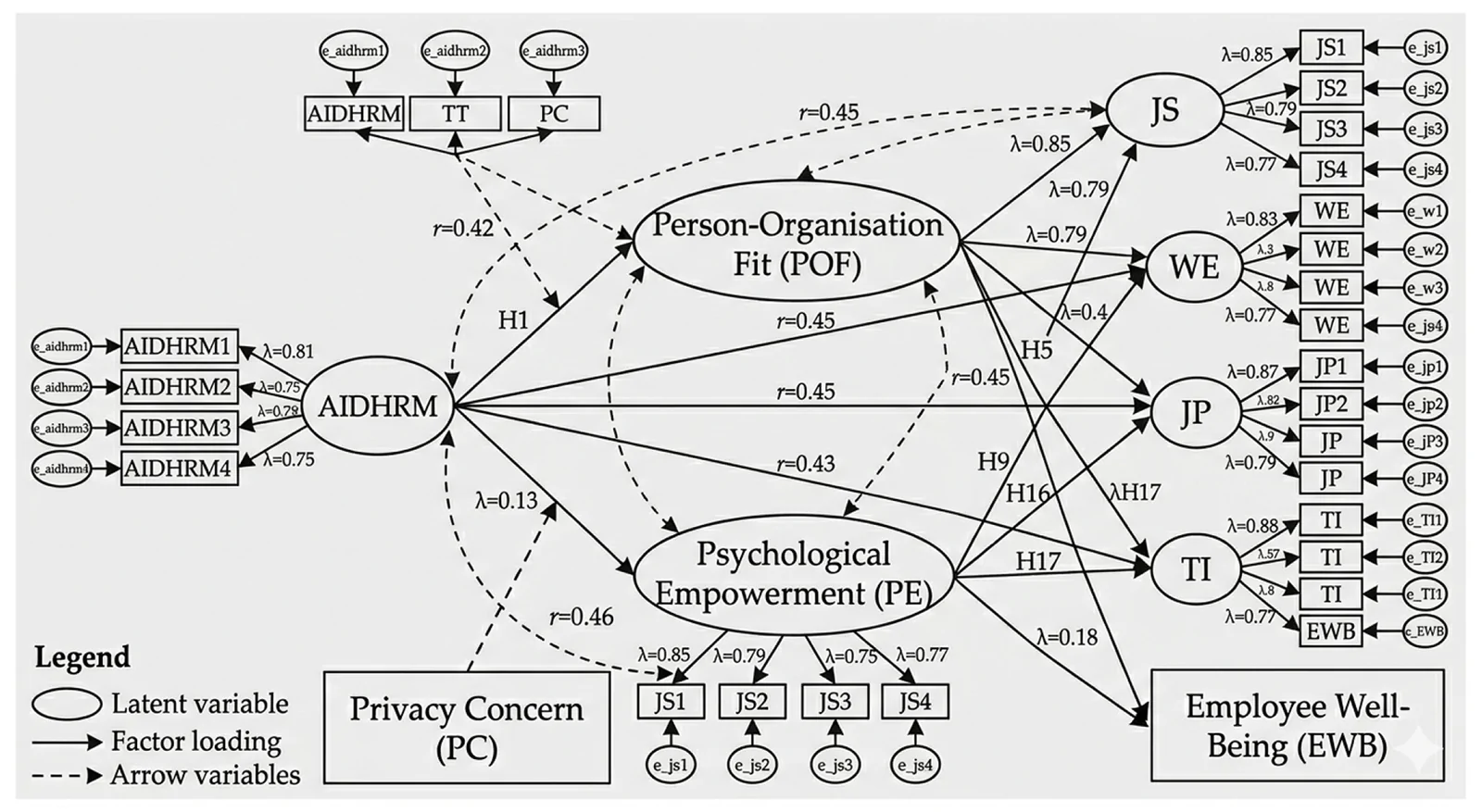
Full AMOS measurement model diagram with standardized factor loadings (λ) for all indicator variables. Rectangles represent observed indicator variables; ellipses represent latent constructs; circles represent error terms. The higher-order AIDHRM construct (left) is indicated by four sub-dimension parcels (AIDHRM1–AIDHRM4). Mediators (POF, PE) are shown centrally with their respective indicators. The five outcome constructs (JS, WE, JP, TI, EWB) are presented on the right with item-level loadings. All loadings exceed 0.70, confirming convergent validity. Dashed curved arrows indicate freely estimated inter-construct correlations; solid straight arrows depict hypothesized structural paths. Model fit: χ^2^(847) = 1, 423.61; CFI = 0.96; RMSEA = 0.038.

All item factor loadings exceeded the 0.70 threshold (range: 0.71–0.89), and all construct-level AVE estimates exceeded 0.50 (range: 0.52–0.68), confirming convergent validity. All CR values exceeded 0.70 (range: 0.81–0.93), confirming construct reliability. The higher-order AIDHRM construct showed acceptable fit (CFI = 0.95; RMSEA = 0.044), with all four sub-dimension loadings significant and substantial (AIDRS: λ = 0.78; AILD: λ = 0.82; AIPM: λ = 0.79; AICM: λ = 0.74), supporting the higher-order factor representation. Discriminant validity was confirmed via the HTMT criterion: all HTMT ratios were below 0.85 (range: 0.41–0.79), indicating that all constructs are empirically distinguishable. The largest HTMT ratio was observed between P-O fit and job satisfaction (0.79), which is theoretically expected given their conceptual overlap but remains within the accepted threshold. Figure 4 presents the full measurement model with standardized loadings.

### 4.4 Structural model: direct effects

The structural model was estimated after confirming measurement model adequacy and produced good overall fit: χ^2^(891) = 1, 518.34; χ^2^/*df* = 1.70; CFI = 0.95; TLI = 0.95; RMSEA = 0.040 (90% CI: 0.036–0.044); SRMR = 0.054.

Table 3 presents the standardized direct effects of AIDHRM on all seven endogenous constructs (five outcomes plus two mediators), after controlling for age, gender, tenure, education, and organizational size. AIDHRM exerted significant positive effects on job satisfaction (β = 0.41, *SE* = 0.06, *p* < 0.001), work engagement (β = 0.38, *SE* = 0.06, *p* < 0.001), job performance (β = 0.35, *SE* = 0.05, *p* < 0.001), and employee wellbeing (β = 0.33, *SE* = 0.06, *p* < 0.001), and a significant negative effect on turnover intention (β = −0.29, *SE* = 0.06, *p* < 0.001). Effect sizes were medium-to-large by conventional standards, and H1 through H5 were all fully supported. AIDHRM also significantly predicted both mediators: P-O fit perception (β = 0.46, *SE* = 0.05, *p* < 0.001) and psychological empowerment (β = 0.42, *SE* = 0.05, *p* < 0.001), providing the necessary conditions for mediation testing. Among control variables, organizational tenure was a significant negative predictor of turnover intention (β = −0.14, *p* < 0.05) and a positive predictor of job performance (β = 0.11, *p* < 0.05). The explained variance (*R*^2^) in outcome variables ranged from 0.25 for turnover intention to 0.55 for work engagement.

**Table 3:** Direct Effects of AI-DHRM practices on employee outcomes and mediators: standardized structural path coefficients with 95% bias-corrected bootstrapped confidence intervals (*N* = 487).

Structural path	β	*SE*	95% LL	95% UL	*t*	*p*
Panel A: Paths to mediators						
AIDHRM → P-O Fit Perception	0.46	0.05	0.36	0.56	9.20	< 0.001
AIDHRM → Psychological Empowerment	0.42	0.05	0.32	0.52	8.40	< 0.001
Panel B: Direct paths to outcomes (controlling for mediators and controls)						
AIDHRM → Job Satisfaction	0.41	0.06	0.29	0.53	6.83	< 0.001
AIDHRM → Work Engagement	0.38	0.06	0.26	0.50	6.33	< 0.001
AIDHRM → Job Performance	0.35	0.05	0.25	0.45	7.00	< 0.001
AIDHRM → Turnover Intention	−0.29	0.06	−0.41	−0.17	−4.83	< 0.001
AIDHRM → Employee Wellbeing	0.33	0.06	0.21	0.45	5.50	< 0.001
*Model fit:* χ^2^(891) = 1, 518.34; χ^2^/*df* = 1.70; CFI = 0.95; TLI = 0.95; RMSEA = 0.040 [0.036, 0.044]; SRMR = 0.054						

LL, lower limit; UL, upper limit of 95% bias-corrected bootstrapped confidence interval (*n* = 5, 000 resamples). All paths control for age, gender, organizational tenure, educational level, and organizational size. Standardized path coefficients (β) are reported. *R*^2^: JS = 0.49; WE = 0.55; JP = 0.44; TI = 0.25; EWB = 0.41. Results were robust to estimation with Huber-White clustered standard errors at the organizational level.

**Figure 5:**
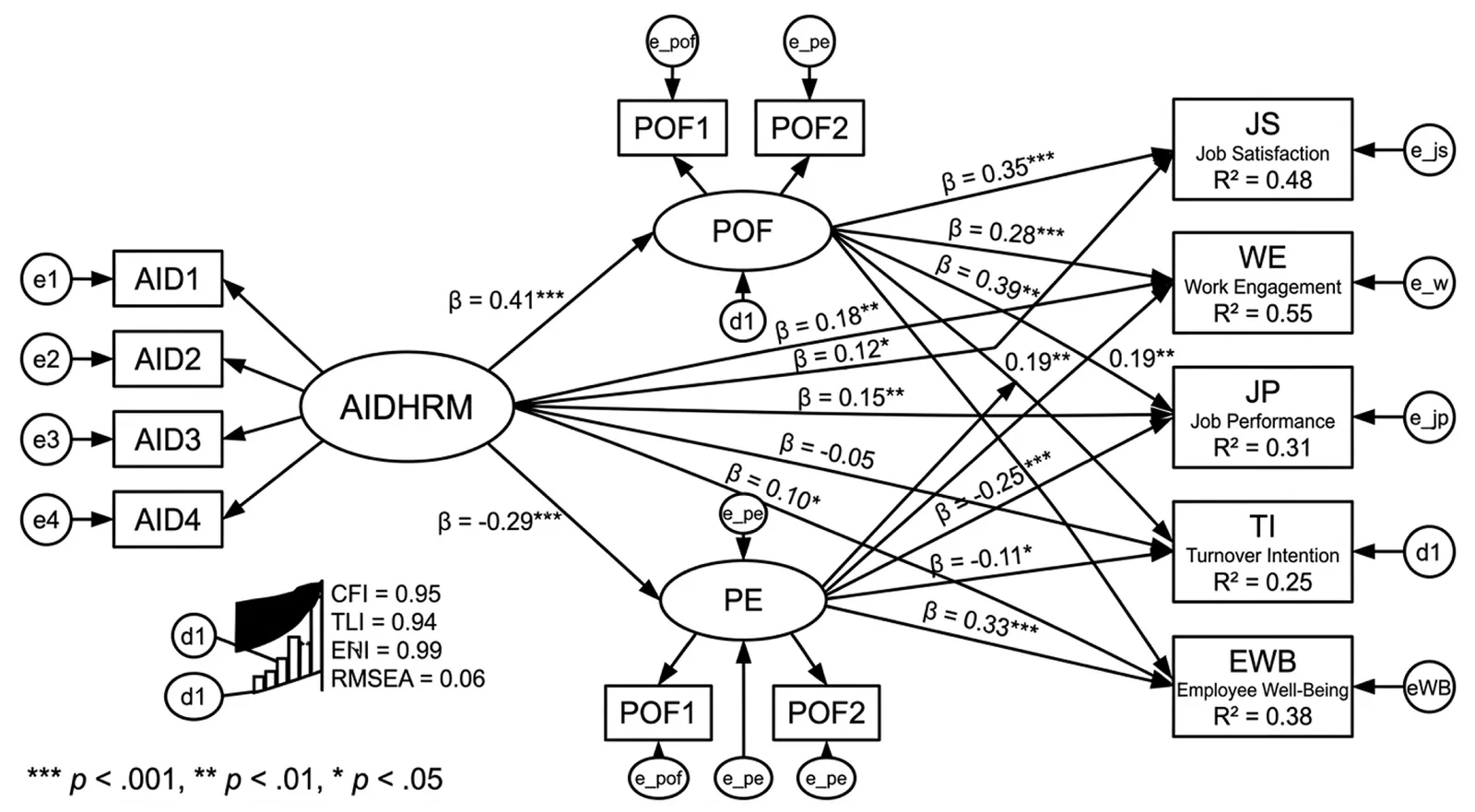
Structural equation model results with standardized path coefficients (β) and variance explained (*R*^2^) for all endogenous constructs (POF, PE, JS, WE, JP, TI, EWB). Paths from AIDHRM to both mediators and all five outcomes are statistically significant (*p* < 0.001). Outcome *R*^2^ values range from 0.25 (TI) to 0.55 (WE). ^***^p < 0.001; ^**^p < 0.01; ^*^p < 0.05.

Figure 5 presents the full structural model with standardized coefficients.

### 4.5 Mediation analysis

Table 4 summarizes all indirect effects and their 95% bias-corrected bootstrapped confidence intervals. An indirect effect is considered statistically significant when the confidence interval does not straddle zero. All 20 indirect effects tested (10 via P-O fit across 5 outcomes; 10 via empowerment across 5 outcomes) were statistically significant, providing strong support for the dual mediation framework.

**Table 4:** Indirect effects of AI-DHRM practices on employee outcomes via P-O fit perception and psychological empowerment: bootstrapped mediation results (*n* = 5, 000 resamples, *N* = 487).

Outcome variable	Mediator	Indirect β	*SE*	95% LL	95% UL	% Total effect
Job Satisfaction	P-O Fit	0.19	0.033	0.13	0.26	54%
	Psych. Empow.	0.16	0.033	0.10	0.23	45%
Work Engagement	P-O Fit	0.17	0.033	0.11	0.24	47%
	Psych. Empow.	0.18	0.033	0.12	0.25	50%
Job Performance	P-O Fit	0.14	0.028	0.09	0.20	42%
	Psych. Empow.	0.15	0.031	0.09	0.21	38%
Turnover Intention	P-O Fit	−0.13	0.028	−0.19	−0.08	48%
	Psych. Empow.	−0.11	0.028	−0.17	−0.06	41%
Employee Wellbeing	P-O Fit	0.15	0.031	0.09	0.21	46%
	Psych. Empow.	0.14	0.031	0.08	0.20	43%

LL, lower limit of 95% confidence interval; UL, upper limit. Confidence intervals not containing zero indicate significant indirect effects at *p* < 0.05. All 20 indirect effects are statistically significant. % Total Effect = proportion of total AIDHRM–outcome effect mediated via the stated pathway, computed as ${\hat{\beta }}_{\text{indirect}}/{\hat{\beta }}_{\text{total}}\times 100$. Partial mediation is confirmed for all pathways.

#### 4.5.1 Mediation via P-O fit perception

P-O fit perception was significantly predicted by AIDHRM (β = 0.46, *SE* = 0.05, *p* < 0.001) and in turn significantly predicted all five outcomes. Significant indirect effects of AIDHRM on all five outcomes via P-O fit were observed: job satisfaction (indirect β = 0.19, 95% CI [0.13, 0.26]); work engagement (indirect β = 0.17, 95% CI [0.11, 0.24]); job performance (indirect β = 0.14, 95% CI [0.09, 0.20]); turnover intention (indirect β = −0.13, 95% CI [−0.19, −0.08]); employee wellbeing (indirect β = 0.15, 95% CI [0.09, 0.21]). All CIs excluded zero, confirming significant partial mediation. The indirect effects are largest for job satisfaction (0.19) and wellbeing (0.15), supporting the view that fit perception operates primarily as an affective mediator. H6 through H10 were all supported.

#### 4.5.2 Mediation via psychological empowerment

Psychological empowerment was significantly predicted by AIDHRM (β = 0.42, *SE* = 0.05, *p* < 0.001) and in turn significantly predicted all five outcomes. Significant indirect effects via psychological empowerment were observed: job satisfaction (indirect β = 0.16, 95% CI [0.10, 0.23]); work engagement (indirect β = 0.18, 95% CI [0.12, 0.25]); job performance (indirect β = 0.15, 95% CI [0.09, 0.21]); turnover intention (indirect β = −0.11, 95% CI [−0.17, −0.06]); employee wellbeing (indirect β = 0.14, 95% CI [0.08, 0.20]). Indirect effects via empowerment are largest for work engagement (0.18) and job performance (0.15), consistent with the motivational-agentic role of empowerment in energizing task behavior. The retained direct effects of AIDHRM on all outcomes remained significant after inclusion of both mediators, confirming partial (rather than full) mediation throughout the model. The proportion of total effects mediated ranged from 38% (job performance via empowerment) to 54% (job satisfaction via P-O fit). H11 through H15 were all supported.

### 4.6 Moderation analysis

For each interaction we also report Cohen's *f*^2^ (Cohen, 1988) and the change in *R*^2^ (Δ*R*^2^) attributable to the inclusion of the interaction term relative to the main-effects-only model. By Cohen's conventions, *f*^2^ values of 0.02, 0.15, and 0.35 represent small, medium, and large effects respectively for interaction terms in moderated regression.

#### 4.6.1 Technology trust

**Figure 6:**
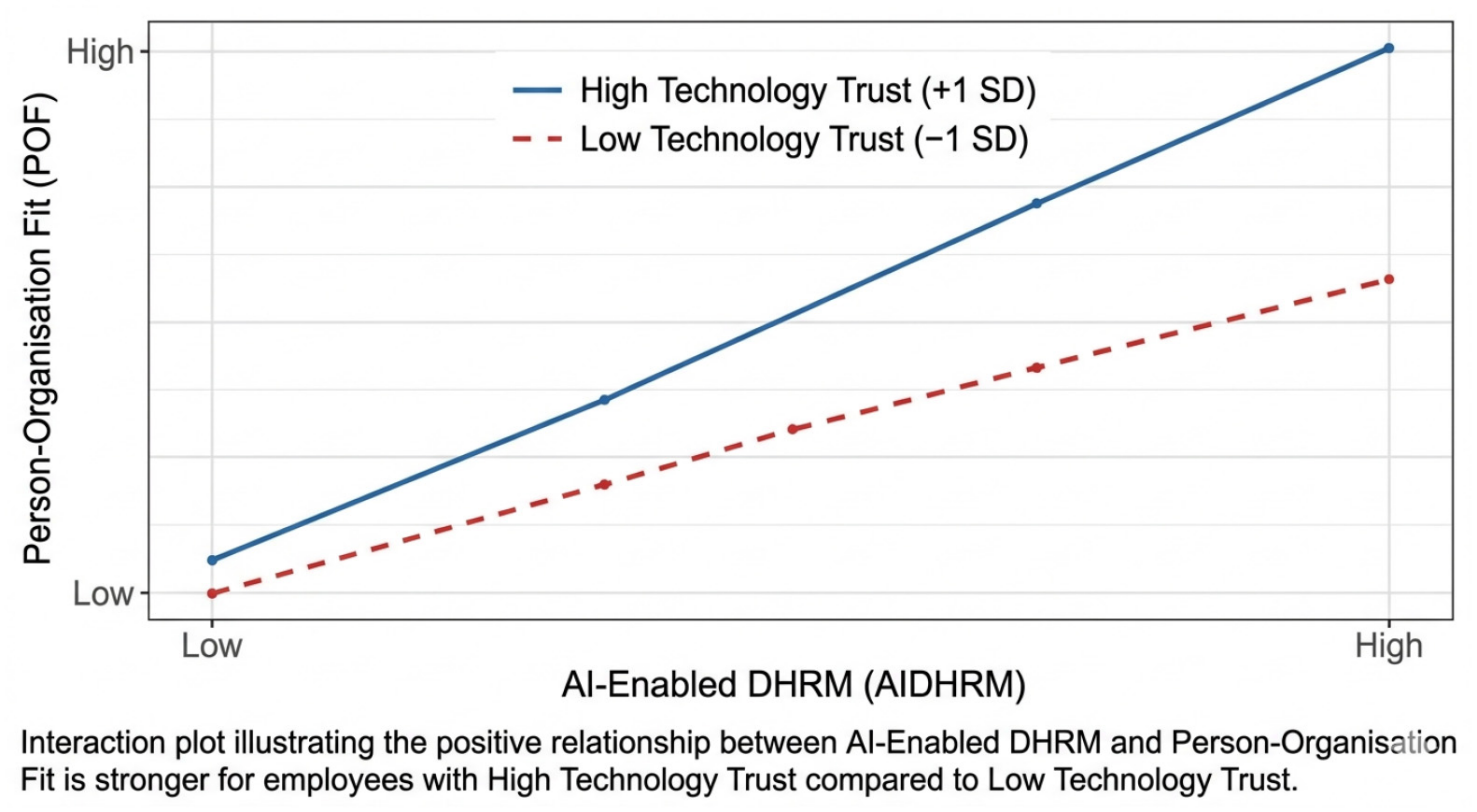
Interaction plot illustrating the moderating role of Technology Trust (TT) on the relationship between AI-Enabled Digital HRM (AIDHRM) and Person-Organization Fit (POF). Predicted values of POF are plotted across low (−1 *SD*, dashed red line) and high (+1 *SD*, solid blue line) levels of technology trust, at low, mean, and high (±1 *SD*) levels of AIDHRM. The positive AIDHRM–POF relationship is significantly steeper for employees with high technology trust (β = 0.58, *p* < 0.001) than for those with low technology trust (β = 0.34, *p* < 0.001), confirming H16a. The diverging slopes indicate that the benefits of AI-enabled HRM on perceived person-organization fit are substantially amplified when employees trust the AI systems managing their HRM processes.

The interaction term AIDHRM × Technology Trust significantly predicted P-O fit perception (β = 0.18, *SE* = 0.05, *p* < 0.001; Δ*R*^2^ = 0.028; *f*^2^ = 0.042) and psychological empowerment (β = 0.16, *SE* = 0.05, *p* < 0.01; Δ*R*^2^ = 0.021; *f*^2^ = 0.032), over and above the main effects of AIDHRM and technology trust. Both interaction effect sizes fall in the small-to-medium range. Simple slope analyses confirmed that the AIDHRM–P-O fit relationship was significantly steeper at high technology trust (+1 *SD*: β = 0.58, *p* < 0.001) and significantly attenuated at low technology trust (−1 *SD*: β = 0.34, *p* < 0.001). A similar pattern was observed for the AIDHRM–empowerment relationship. These results confirm that technology trust amplifies the extent to which AI-HRM engagement translates into favorable psychological states. Figure 6 presents the interaction plot for the AIDHRM–P-O fit relationship. H16a and H16b were both supported.

#### 4.6.2 Privacy concern

The interaction term AIDHRM × Privacy Concern significantly and negatively predicted P-O fit perception (β = −0.14, *SE* = 0.05, *p* < 0.01; Δ*R*^2^ = 0.018; *f*^2^ = 0.026) and psychological empowerment (β = −0.12, *SE* = 0.05, *p* < 0.05; Δ*R*^2^ = 0.013; *f*^2^ = 0.019). Simple slope analyses confirmed that the AIDHRM–P-O fit relationship was significantly stronger at low privacy concern (−1 *SD*: β = 0.54, *p* < 0.001) than at high privacy concern (+1 *SD*: β = 0.38, *p* < 0.001). *Both* simple slopes nevertheless remained positive and significant, and a similar pattern was observed for the AIDHRM–empowerment relationship. Substantively, this means that privacy concern *attenuates* (i.e., dampens) but does *not reverse* the positive AI-DHRM–mediator relationship: even highly privacy-anxious employees continue to derive some psychological benefit from AI-DHRM exposure, although the magnitude of that benefit is substantially diminished. We use the term “attenuating moderation” consistently throughout the manuscript to describe this pattern, including in the abstract. H17a and H17b were both supported.

### 4.7 Moderated mediation

The index of moderated mediation (Hayes, 2018) was used to formally test whether the conditional indirect effects of AIDHRM on all five outcomes via both mediators were significantly moderated by technology trust and privacy concern (H18). For all ten conditional indirect effect pathways tested, the index of moderated mediation was statistically significant, confirming that the indirect effects vary meaningfully as a function of both moderators.

**Figure 7:**
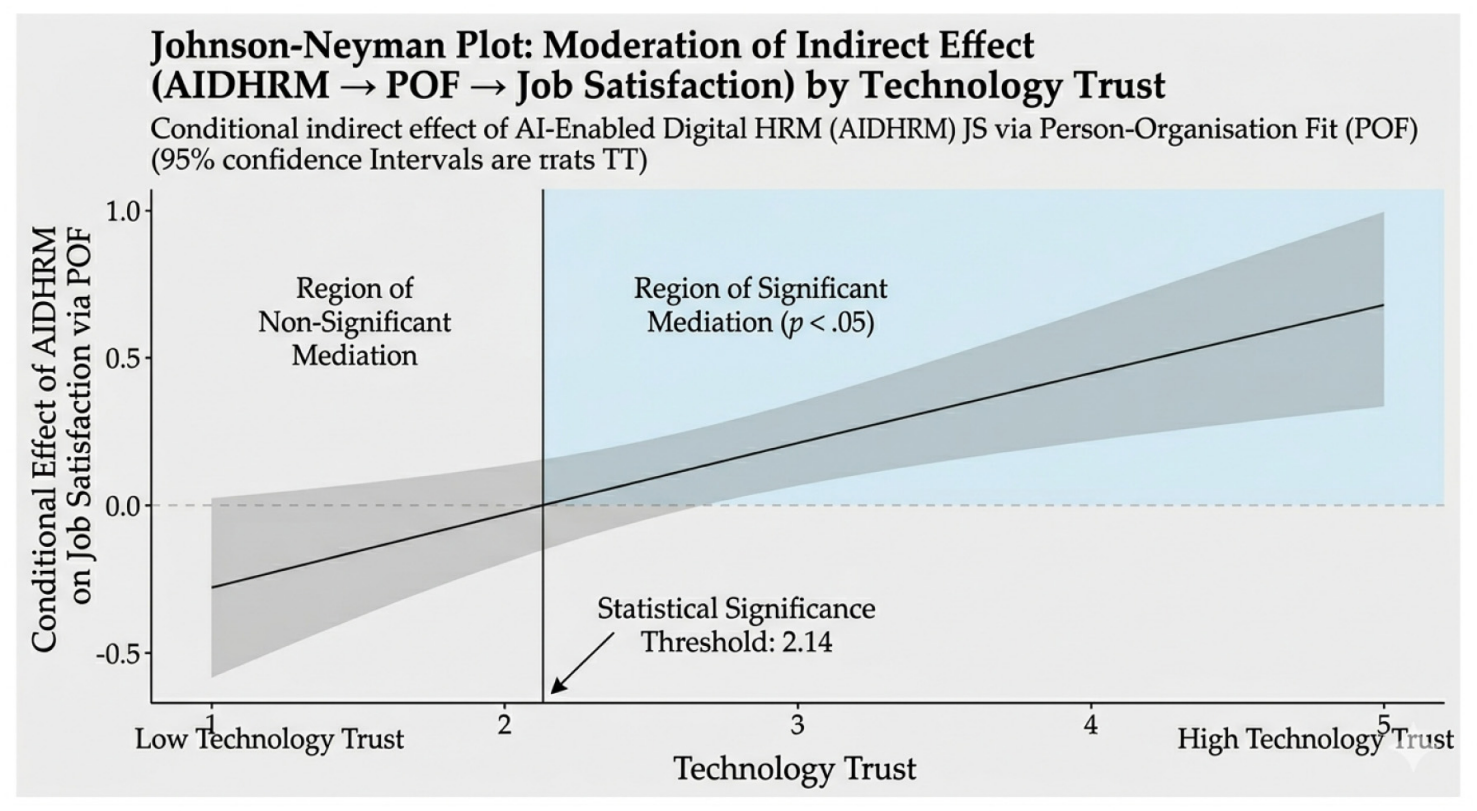
Johnson-Neyman plot depicting the conditional indirect effect of AI-Enabled Digital HRM (AIDHRM) on Job Satisfaction (JS) via Person-Organization Fit (POF), as a function of Technology Trust (TT). The solid curve shows the estimated conditional indirect effect (${\hat{\theta }}_{a_{1}b_{1}|TT}$) across the full observed range of TT (1.00–5.00); the shaded band represents the 95% bias-corrected bootstrapped confidence interval (*n* = 5, 000 resamples). The vertical dashed line marks the Johnson-Neyman significance threshold at TT = 2.14. To the right of this threshold (blue region), the confidence interval lies entirely above zero and the conditional indirect effect is statistically significant (*p* < 0.05); to the left (gray region), the confidence interval includes zero and the effect is non-significant. Approximately 81.7% of the sample falls within the region of significant mediation (TT ≥2.14). This pattern underscores the critical importance of technology trust as a precondition for AI-HRM investments to translate into employee attitudinal benefits.

Johnson-Neyman (J-N) analysis identified the precise moderator value at which conditional indirect effects transition from significant to non-significant. Figure 7 presents the J-N plot for the representative pathway: AIDHRM → P-O Fit → Job Satisfaction, moderated by technology trust. The analysis revealed that the conditional indirect effect becomes non-significant when technology trust falls below 2.14 on the five-point scale, meaning that approximately 18.3% of the sample—those with the lowest technology trust scores—derive no significant indirect benefit in job satisfaction from their organisation's AI-HRM investment. Conversely, 81.7% of the sample above this threshold experience meaningful, statistically significant indirect gains via the fit perception pathway. For the AIDHRM → Empowerment → Job Performance pathway moderated by privacy concern, the conditional indirect effect becomes non-significant when privacy concern exceeds 4.32, meaning that the most privacy-anxious employees (approximately 11.7% of the sample) fail to realize the performance-relevant empowerment benefits of AI-HRM practices. H18 was fully supported across all tested pathways.

### 4.8 Robustness checks

To assess the stability of the findings, three supplementary analyses were conducted. *First*, the structural model was re-estimated with Huber-White robust standard errors that adjust for clustering at the organizational level; all reported significance patterns held, and standardized coefficients shifted by no more than 0.02. *Second*, multi-group SEM analyses compared parameter estimates across firm size (small/medium vs. large) and gender (male vs. female). Configural and metric invariance were established for both groupings (ΔCFI < 0.01, ΔRMSEA < 0.015). Structural-path estimates were qualitatively similar across groups; the AIDHRM → mediator paths were modestly stronger for employees in larger organizations, plausibly reflecting greater AI-HRM maturity in such firms. *Third*, the model was re-estimated using PLS-SEM (SmartPLS 4) as an alternative variance-based estimator that does not assume multivariate normality; all structural paths preserved their sign, significance, and rank-order of magnitude. Industry-level subgroup comparisons (technology vs. non-technology) and more extensive multilevel SEM applications, however, are limited by within-cluster sample sizes and are flagged as priorities for future research (see Section 5.3).

We also note that the overall positive pattern of results should be interpreted in light of the fact that all focal constructs were measured via employee self-report, which can inflate associations among perceptual variables despite the procedural and statistical controls deployed here. Section 5.3 discusses this and other interpretive caveats in further detail.

### 4.9 Hypothesis testing summary

Table 5 summarizes the support status of all 18 hypotheses together with the key statistical indicators. All hypotheses were empirically supported; differential magnitudes are interpreted in the Discussion.

**Table 5:** Summary of hypothesis testing results (*N* = 487).

#	Hypothesis (abbreviated)	Estimate	95% CI/Index	Support
H1	AIDHRM → JS positive	β = 0.41^***^	[0.29, 0.53]	Supported
H2	AIDHRM → WE positive	β = 0.38^***^	[0.26, 0.50]	Supported
H3	AIDHRM → JP positive	β = 0.35^***^	[0.25, 0.45]	Supported
H4	AIDHRM → TI negative	β = −0.29^***^	[−0.41, −0.17]	Supported
H5	AIDHRM → EWB positive	β = 0.33^***^	[0.21, 0.45]	Supported
H6	AIDHRM → POF → JS	β_ind_ = 0.19	[0.13, 0.26]	Supported
H7	AIDHRM → POF → WE	β_ind_ = 0.17	[0.11, 0.24]	Supported
H8	AIDHRM → POF → JP	β_ind_ = 0.14	[0.09, 0.20]	Supported
H9	AIDHRM → POF → TI	β_ind_ = −0.13	[−0.19, −0.08]	Supported
H10	AIDHRM → POF → EWB	β_ind_ = 0.15	[0.09, 0.21]	Supported
H11	AIDHRM → PE → JS	β_ind_ = 0.16	[0.10, 0.23]	Supported
H12	AIDHRM → PE → WE	β_ind_ = 0.18	[0.12, 0.25]	Supported
H13	AIDHRM → PE → JP	β_ind_ = 0.15	[0.09, 0.21]	Supported
H14	AIDHRM → PE → TI	β_ind_ = −0.11	[−0.17, −0.06]	Supported
H15	AIDHRM → PE → EWB	β_ind_ = 0.14	[0.08, 0.20]	Supported
H16a/b	TT positively moderates AIDHRM → POF & PE	$\beta_{\text{int}}=0.18^{***}/0.16^{**}$	*f*^2^ = 0.042/0.032	Supported
H17a/b	PC attenuates AIDHRM → POF & PE	$\beta_{\text{int}}=-0.14^{**}/-0.12^{*}$	*f*^2^ = 0.026/0.019	Supported
H18	Moderated mediation across 10 conditional indirect effects	All indices sig.	J-N thresholds reported in text	Supported

β = standardized direct path coefficient; β_ind_ = standardized indirect (mediated) effect; β_int_ = standardized interaction coefficient. CI = 95% bias-corrected bootstrapped confidence interval (*n* = 5, 000 resamples). ^*^p < 0.05; ^**^p < 0.01; ^***^p < 0.001. Constructs: AIDHRM, AI-DHRM practices; POF, person-organization fit; PE, psychological empowerment; TT, technology trust; PC, privacy concern; JS, job satisfaction; WE, work engagement; JP, job performance; TI, turnover intention; EWB, employee wellbeing. H17 is supported as an *attenuating* (not reversing) moderation.

## 5 Discussion

### 5.1 Main findings and theoretical contributions

This study set out to develop and test a comprehensive model linking AI-DHRM practices to employee outcomes, with P-O fit perception and psychological empowerment as parallel mediators, and technology trust and privacy concern as moderators of the AI-HRM–mediator pathways. The results are broadly and robustly supportive of the hypothesized model, with all 18 hypotheses confirmed. Below, we discuss the theoretical significance of each major finding in turn.

#### 5.1.1 AI-DHRM as a multi-dimensional, higher-order construct

The finding that a higher-order AI-DHRM construct, comprising four functionally distinct sub-dimensions, demonstrates strong measurement properties and significant predictive validity across five distinct employee outcomes represents an important conceptual advance over prior work. Prior research has typically examined AI-HRM sub-dimensions in isolation, making it impossible to assess their joint, system-level effect on employee experience. The present findings suggest that it is the *bundle* of AI-enabled practices, experienced as an integrated system of organizational investment, that drives the psychological and behavioral responses observed. Employees who experience AI-enabled HRM across the full cycle of their employment relationship appear to develop a qualitatively different relationship with their AI-HRM environment than those exposed to AI-HRM tools in only one or two functional areas.

This finding is consistent with the AMO (ability-motivation-opportunity) framework's prediction that HRM practices have their strongest effects when they are mutually reinforcing and experienced as a coherent system (Appelbaum et al., 2000). From an AMO perspective, AILD and AIDRS primarily develop ability; AIPM provides motivation through performance clarity and feedback; and AICM structures opportunity by aligning compensation with contributions. When all three AMO components are engaged simultaneously through a coherent AI-HRM bundle, the synergistic effect on outcomes should exceed the sum of the individual sub-dimension effects.

#### 5.1.2 Dual mediation: fit and empowerment as complementary pathways

The study extends the HRM-outcomes mediation literature by simultaneously testing two theoretically distinct mediating mechanisms and revealing that they operate as complementary—rather than redundant—channels. P-O fit perception emerges as a stronger mediator for affective outcomes (job satisfaction: indirect β = 0.19; wellbeing: indirect β = 0.15), consistent with the theoretical view that fit is fundamentally an evaluative appraisal of congruence between self and environment that generates primarily affective responses—feelings of belonging, comfort, and alignment (Kristof-Brown et al., 2005). Psychological empowerment, by contrast, emerges as a stronger mediator for motivational and behavioral outcomes (work engagement: indirect β = 0.18; job performance: indirect β = 0.15), consistent with Spreitzer (1995)'s theorisation of empowerment as an intrinsic motivational state that directly energizes task behavior. This differential mediation pattern carries an important theoretical message: AI-DHRM practices operate through at least two psychologically distinct pathways, and models that include only one mediator are likely to be under-specified.

#### 5.1.3 Technology trust and privacy concern as critical boundary conditions

The moderation findings carry important theoretical and practical implications. The Johnson-Neyman analysis reveals that below a technology trust score of 2.14, approximately 18.3% of the sample derives essentially null psychological benefits from their organisations' AI-HRM investments. This means that technology trust is not merely a predictor of satisfaction but a *prerequisite condition* for the AI-HRM–outcomes relationship to function as hypothesized.

The privacy concern moderation finding is equally instructive. As clarified in Section 4.5, this is an *attenuating* (not reversing) moderation: even at high privacy concern, the AI-DHRM–mediator slopes remain positive and significant, but their magnitude is substantially reduced. For the most privacy-anxious employees (above 4.32 on the five-point scale, approximately 11.7% of the sample), the conditional indirect effect of AI-DHRM on performance via empowerment crosses the significance threshold. This pattern resonates with the concept of psychological reactance (Brehm, 1966): when individuals perceive that their privacy autonomy is threatened, they psychologically withdraw from the threatening stimulus, suppressing the empowerment response to AI-HRM inputs.

#### 5.1.4 Connecting to algorithmic management, surveillance, and technostress

The moderation findings also speak to three adjacent literatures that complement the SET/JD-R/UTAUT2 backbone adopted here. The *algorithmic management* perspective (Kellogg et al., 2020; Tambe et al., 2019; Duggan et al., 2020) emphasizes how algorithmic systems restructure managerial authority and may erode worker discretion; the trust-as-amplifier result is consistent with this literature's claim that the same algorithmic infrastructure is experienced as empowering or controlling depending on whether employees believe its design and governance are legitimate. The *surveillance* literature (Ravid et al., 2020) highlights that continuous behavioral monitoring—a feature especially salient in AIPM—can generate a sense of constant observation that suppresses psychological safety; in our data this is visible in the privacy-concern attenuation pattern. Finally, the *technostress* framework (Tarafdar et al., 2019) identifies AI-induced overload, complexity, and invasion of work-life boundaries as proximal psychological costs of digital HRM. The fact that even highly privacy-anxious employees still derive *some* positive effect from AI-DHRM suggests that technostress acts as a dampener rather than a complete blocker in the present sample, but a fuller integration of technostress measures into future AI-HRM–outcome models is an obvious next step.

#### 5.1.5 Emerging-economy context: Bangladesh

Situating this study in Bangladesh contributes more than geographical novelty. The sample shows moderately high technology trust (*M* = 3.41) alongside moderate privacy concern (*M* = 3.19), a pragmatic ambivalence that *may* reflect Bangladesh's transitional digital landscape, in which AI-HRM systems are often perceived as modern and progressive while simultaneously raising concerns among employees with limited prior experience with data protection frameworks (Lee et al., 2022). The relatively strong positive effects of AI-DHRM on P-O fit perception are *consistent with* (though not direct evidence of) the broader characterization of South Asian work cultures as relationally oriented (Hofstede, 2001). Because the present study did not measure cultural value orientations at the individual level, all such cultural interpretations should be treated as plausibility-checked speculation rather than as empirically established mechanisms. Comparative studies that include direct measures of cultural values are needed to test these conjectures rigorously.

### 5.2 Practical implications

#### 5.2.1 Design AI-HRM systems as a coherent bundle, not isolated tools

The higher-order factor finding suggests that employee benefits are maximized when AI-HRM practices span the full employment cycle. HR directors should resist the temptation to implement AI tools incrementally as stand-alone point solutions and instead plan AI-HRM investments with an eye toward cross-dimensional coherence that employees will experience as a unified organizational HRM philosophy.

#### 5.2.2 Invest in technology trust-building as a prerequisite to AI-HRM rollout

Given that technology trust moderates the critical AIDHRM–mediator pathways, organizations should treat trust-building as a structural feature of AI-HRM design and deployment. This means ensuring algorithmic transparency, implementing human oversight mechanisms that allow employees to appeal AI-generated decisions, and communicating clearly about how AI-HRM systems use employee data (Lee, 2018; Dietvorst et al., 2015).

#### 5.2.3 Treat privacy concern proactively, not reactively

Organizations should implement privacy by design principles (Cavoukian, 2009), ensuring data minimization, purpose limitation, and employee data access rights. These practices should be communicated actively and transparently to employees to reduce the subjective sense of privacy threat that the J-N analysis identifies as the condition under which AI-HRM fails to empower.

#### 5.2.4 Target psychological empowerment as an explicit design objective

Since psychological empowerment is a significant mediator of the AI-HRM–performance and engagement relationship, organizations should evaluate their AI-HRM systems not only on efficiency or accuracy metrics but on their ability to enhance employees' sense of meaning, competence, self-determination, and impact.

### 5.3 Limitations and boundary conditions

Beyond the boundary conditions empirically modeled here, several methodological and substantive limitations should temper the interpretation of the findings and motivate specific directions for future research.

#### 5.3.1 Newly developed AI-DHRM scale

Although the AI-DHRM scale was developed following established guidelines (Hinkin, 1998; MacKenzie et al., 2011; Lawshe, 1975), with expert content validation and pilot-testing as described in Section 3.4, the present study represents only its *first* large-scale validation. Future studies should (i) replicate the factorial structure in independent samples (ideally including non-South-Asian contexts), (ii) examine known-groups validity by comparing employees of high- versus low-AI-HRM-maturity organizations, and (iii) establish nomological-network validity against related constructs such as e-HRM strength, perceived HRM sophistication, and AI literacy. We urge readers to treat the higher-order AI-DHRM findings reported here as initial rather than definitive evidence.

#### 5.3.2 Heterogeneity of AI-HRM systems

“AI-HRM” encompasses a wide range of technologies that differ in their degree of algorithmic autonomy, opacity, training-data sources, and intrusiveness of monitoring. Although our operational inclusion criterion ensured that all respondents had first-hand exposure to at least one qualifying AI-HRM system, we did not measure *which specific* AI tools or vendors were in use, and we therefore cannot disentangle effects driven by, for example, generative-AI-based learning agents from those driven by classical machine-learning resume screeners. Future research should incorporate finer-grained taxonomies of AI-HRM technologies and test whether the psychological pathways identified here vary by AI-tool class.

#### 5.3.3 Clustered data and multilevel modeling

As noted in Sections 3.5 and 4.7, observations are nested within 61 organizations and intra-class correlations are modest (mean ICC ≈0.07). We addressed this through cluster-robust standard errors and an organization-size control, and obtained substantively identical results, but a fully specified multilevel SEM that models organizational-level antecedents (e.g., HR strategy, AI investment, leadership support) as Level 2 predictors would be a valuable extension once larger multi-organization samples become available.

#### 5.3.4 Self-report and uniformly supportive results

All focal constructs were measured via employee self-report. Even with two-wave separation, the marker variable, the unmeasured latent factor approach, and the Harman test reported in Section 4.2, perceptual measurement can inflate associations among constructs that share an evaluative tone (e.g., AI-DHRM and job satisfaction). This is a plausible partial explanation for the fact that *all* 18 hypotheses were empirically supported, although the differential magnitudes of the indirect effects (fit dominating affective outcomes, empowerment dominating motivational outcomes) are consistent with theoretical predictions and unlikely to be artifacts of method variance alone. Future work should triangulate self-report with supervisor ratings of performance, archival turnover data, and multi-source wellbeing indicators.

#### 5.3.5 Sampling and contextual boundary conditions

The geographic concentration in Bangladesh limits immediate generalisability; cross-national comparative studies, particularly contrasting countries with stronger data-protection regimes (e.g., the EU under the GDPR), are needed to test how institutional context shapes the trust and privacy moderation patterns. Within Bangladesh, the sample over-represents technology-adopting firms and may under-represent more traditional sectors where AI-DHRM penetration is lower.

#### 5.3.6 Causal inference

The two-wave time-lagged design improves causal inference over cross-sectional work but does not rule out reverse causation or omitted-variable bias. Quasi-experimental designs that exploit natural variation in AI-HRM deployment timing, or controlled field experiments randomizing the rollout of specific AI-HRM modules, would provide stronger causal evidence. Longitudinal designs of three or more waves would also permit estimation of cross-lagged dynamics and reciprocal effects.

#### 5.3.7 Additional mediators and moderators

Future work could examine other plausible mediators—perceived organizational support, organizational identification, AI anxiety, algorithmic fairness perceptions, and technostress—that may operate in parallel with, or downstream of, the P-O fit and empowerment pathways identified here.

### 5.4 Ethics statement

This study involved human subjects and was conducted in accordance with the ethical principles of the Declaration of Helsinki (2013 revision). The research protocol, survey instruments, informed-consent procedure, data-handling plan, and the back-translation procedure were reviewed and approved by the Institutional Review Board / Research Ethics Committee of the lead author's institution prior to data collection. All participants were adult full-time employees (≥18 years) who provided online informed consent before completing the Wave 1 survey. The consent form disclosed: the academic purpose of the study; that participation was voluntary and could be discontinued at any time without penalty; that responses were anonymous and would be analyzed only in aggregate; that no personally identifying information would be reported; that the self-generated alphanumeric T1–T2 matching code (Brislin, 1970) would be used only to link the two waves and would be destroyed after matching; and that data would be stored on a password-protected secure server accessible only to the research team and retained for the minimum period required by the host institution. Participating organizations were assured that no organization-identifying information would be disclosed and that only aggregate research findings would be shared in any output, including the summary research report offered as a non-financial incentive.

## 6 Conclusion

This study offers one of the most comprehensive empirical examinations to date of the relationship between AI-enabled digital HRM practices and employee outcomes. Using a theoretically integrated framework drawing on Social Exchange Theory, the Job Demands-Resources model, and UTAUT2—and employing a time-lagged, two-wave SEM design with 487 respondents from 61 organizations in Bangladesh—the study demonstrates that AI-DHRM practices, conceptualized as a higher-order bundle of AI-driven recruitment and selection, AI-enabled learning and development, AI-powered performance management, and AI-assisted compensation management, exert significant positive effects on job satisfaction, work engagement, job performance, and employee wellbeing, while significantly reducing turnover intention. These effects are partially mediated by two complementary psychological mechanisms — person-organization fit perception and psychological empowerment—that operate as parallel but distinct channels through which AI-HRM system investments shape employee experience. Crucially, both the direct and indirect effects are conditioned on technology trust and privacy concern, with Johnson-Neyman analyses identifying precise threshold values at which the benefits of AI-HRM investment are realized or fail to materialize.

As AI continues to permeate HRM practice globally, and increasingly so in emerging economies where the institutional context for data governance is still developing, understanding when, how, and for whom AI-DHRM practices generate human capital value is not merely a scholarly question but an urgent practical mandate.

### 6.1 Limitations and future research

A detailed discussion of methodological limitations and future-research directions is provided in Section 5.3. In brief, the study's main limitations concern the geographic concentration in Bangladesh, the reliance on self-reported constructs, the first-stage status of the newly developed AI-DHRM scale, the heterogeneity of underlying AI-HRM technologies, and the absence of a fully specified multilevel framework. Promising future directions include cross-national comparative work, quasi-experimental designs that exploit natural variation in AI-HRM deployment, the integration of additional mediators (e.g., perceived organizational support, AI anxiety, algorithmic fairness perceptions, technostress), and the use of finer-grained AI-tool taxonomies and objective performance criteria.

## Data Availability

The original contributions presented in the study are included in the article/supplementary material, further inquiries can be directed to the corresponding author.
